# Integrated and Binder‐Free Air Cathodes of Co_3_Fe_7_ Nanoalloy and Co_5.47_N Encapsulated in Nitrogen‐Doped Carbon Foam with Superior Oxygen Reduction Activity in Flexible Aluminum‐Air Batteries

**DOI:** 10.1002/advs.202000747

**Published:** 2020-08-05

**Authors:** Min Jiang, Chaopeng Fu, Ruiqi Cheng, Wei Zhang, Tongyao Liu, Ruibin Wang, Jiao Zhang, Baode Sun

**Affiliations:** ^1^ School of Materials Science and Engineering Shanghai Jiao Tong University Shanghai 200240 P. R. China; ^2^ Advanced Technology Institute University of Surrey Guildford GU2 7XH UK; ^3^ Instrumental Analysis Center of SJTU Shanghai Jiao Tong University Shanghai 200240 P. R. China

**Keywords:** Al‐air batteries, flexible batteries, integrated air cathodes, oxygen reduction

## Abstract

All‐solid‐sate Al‐air batteries with features of high theoretical energy density, low cost, and environmental‐friendliness are promising as power sources for next‐generation flexible and wearable electronics. However, the sluggish oxygen reduction reaction (ORR) and poor interfacial contact in air cathodes cause unsatisfied performance. Herein, a free‐standing Co_3_Fe_7_ nanoalloy and Co_5.47_N encapsulated in 3D nitrogen‐doped carbon foam (Co_3_Fe_7_@Co_5.47_N/NCF) is prepared as an additive‐free and integrated air cathode for flexible Al‐air batteries in both alkaline and neutral electrolytes. The Co_3_Fe_7_@Co_5.47_N/NCF outperforms commercial platinum/carbon (Pt/C) toward ORR with an onset potential of 1.02 V and a positive half‐wave potential of 0.92 V in an alkaline electrolyte (0.59 V in sodium chloride solution), which is ascribed to the unique interfacial structure between Co_3_Fe_7_ and Co_5.47_N supported by 3D N‐doped carbon foam to facilitate fast electron and mass transfer. The high ORR performance is also supported by in*‐*situ electrochemical Raman spectra and density functional theory calculation. Furthermore, the fabricated Al‐air battery displays good flexibility and delivers a power density of 199.6 mW cm^−2^, and the binder‐free and integrated cathode shows better discharge performance than the traditionally slurry casting cathode. This work demonstrates a facile and efficient approach to develop integrated air cathode for metal‐air batteries.

## Graphical abstract

A free‐standing Co_3_Fe_7_ and Co_5.47_N encapsulated in 3D N‐doped carbon foam (Co_3_Fe_7_@Co_5.47_N/NCF) is fabricated and served as an additive‐free and integrated air cathode for flexible Al‐air batteries in both alkaline and neutral electrolytes. The designed air cathode with faster mass and electron transfer demonstrates better battery performance than the traditional slurry casting cathode or commercial Pt/C cathode.

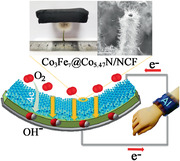

## 1 Introduction

With the increasing interests in developing flexible and wearable electronics, there is an urgent need to develop high performance and low cost power sources for smart electronics.^[^ ^1^, ^2^ ^]^ Metal‐air batteries, especially aluminum‐air (Al‐air) batteries have been considered as a promising alternative because of high theoretical energy density (2796 Wh kg^−1^), low cost and environmental‐friendliness with the features of abundant reserves, light weight and low cost of Al.^[^ ^3^ ^]^ However, the power density of Al‐air batteries is unsatisfied due to sluggish kinetics of oxygen reduction reaction at air cathodes, hindering the wide commercial application.^[^ ^4^, ^5^ ^]^ There are two main challenges which need to be addressed to boost cathode performance for metal‐air batteries. One is to develop the loaded catalysts with Pt‐comparable performance but lower cost, and the other is the fabrication and configuration of air cathodes. Pt‐based catalysts can effectively accelerate oxygen reduction reaction,^[^ ^6^ ^]^ however, the high cost, poor stability and scarcity hinder the commercial application.^[^ ^7^ ^]^ Therefore, it is urgent to develop efficient and cheap ORR catalysts for Al‐air batteries.^[^ ^8^ ^]^ In past decades, transition metal‐based catalysts for ORR have been extensively investigated to replace precious metal catalysts to lower the cost. However, most of these catalysts suffer from unsatisfactory ORR performance.^[^ ^9^, ^10^ ^]^ Recent studies demonstrate that bimetallic nanoalloys can enhance catalytic activity and stability due to the synergistic effect of bimetallic alloys compared to single metals.^[^ ^11^, ^12^ ^]^ Furthermore, introducing transition metal species into N‐doped carbon materials with good electrical conductivities and large surface areas is an efficient approach to enhance electrocatalytic activity.^[^ ^13^, ^14^ ^]^ Additionally, low‐cost transition metal nitrides with typical metallic behavior and abundant M—N covalent bonds also show promising ORR performance due to effective adsorption of oxygen and efficient electron transfer.^[^ ^15^, ^16^, ^17^ ^]^ Moreover, tuning interfacial structure of heterogeneous catalysts is considered as an effective strategy to enhance intrinsic electrocatalytic activity due to the regulation of adsorption strength of ORR intermediates.^[^ ^18^, ^19^ ^]^ Inspired by these attractive properties, it is expected that catalysts with a combination of interfacial structure of bimetallic alloy and transition metal nitrides can be promising candidates toward ORR.

Furthermore, the configuration and structure of air cathodes, determining the charge and mass transfer rate, play a vital role in battery performance. In other words, oxygen reduction catalysts with high activities cannot guarantee high battery performance of air cathodes. Air cathodes are generally fabricated by slurry casting catalyst powder, carbon black and polymer binder onto current collectors. First, catalysts with nanostructure are easily aggregated during casting to deteriorate electrocatalytic activity. Second, a large amount of additives are needed, which not only increases the weight and cost but also complicates the fabrication procedures. Moreover, the non‐conductive binder may increase the inner resistances of air cathodes.^[^ ^20^ ^]^ Therefore, it is of great interests to fabricate additive‐free and integrated air cathodes with high activity to overcome these challenges for high performance metal‐air batteries. Directly in situ growth of catalysts on conductive substrates is considered as a straightforward approach to fabricate such additive‐free and integrated air cathodes.^[^ ^21^, ^22^ ^]^

With these considerations in mind, we report an effective and scalable approach to fabricate an integrated and binder‐free air cathode of Co_3_Fe_7_ nanoalloy and Co_5.47_N encapsulated in nitrogen‐doped carbon foam (Co_3_Fe_7_@Co_5.47_N/NCF) for flexible Al‐air batteries in both alkaline (potassium hydroxide, KOH) and neutral (sodium chloride, NaCl) electrolytes. The Co_3_Fe_7_ nanoalloy and Co_5.47_N encapsulated in 3D N‐doped carbon foam without the use of ancillary materials can facilitate fast mass transport and electron transfer and enhance electrochemical performance and stability. As a result, the Co_3_Fe_7_@Co_5.47_N/NCF with synergetic interfacial structure exhibits superior ORR activity with an onset potential of 1.02 V and a positive half‐wave potential of 0.92 V in 0.1 m KOH solution (0.59 V in NaCl solution), which is confirmed by in situ electrochemical Raman spectra and density functional theory calculations. The ORR performance is superior to that of commercial 20 wt% Pt/C in both alkaline and neutral electrolytes.^[^ ^23^ ^]^ The integrated and binder‐free Co_3_Fe_7_@Co_5.47_N/NCF air electrode with good flexibility displays much better discharge performance than the traditionally slurry casting air cathodes for both alkaline and neutral Al‐air batteries.

## 2 Results and Discussion

**Figure 1:**
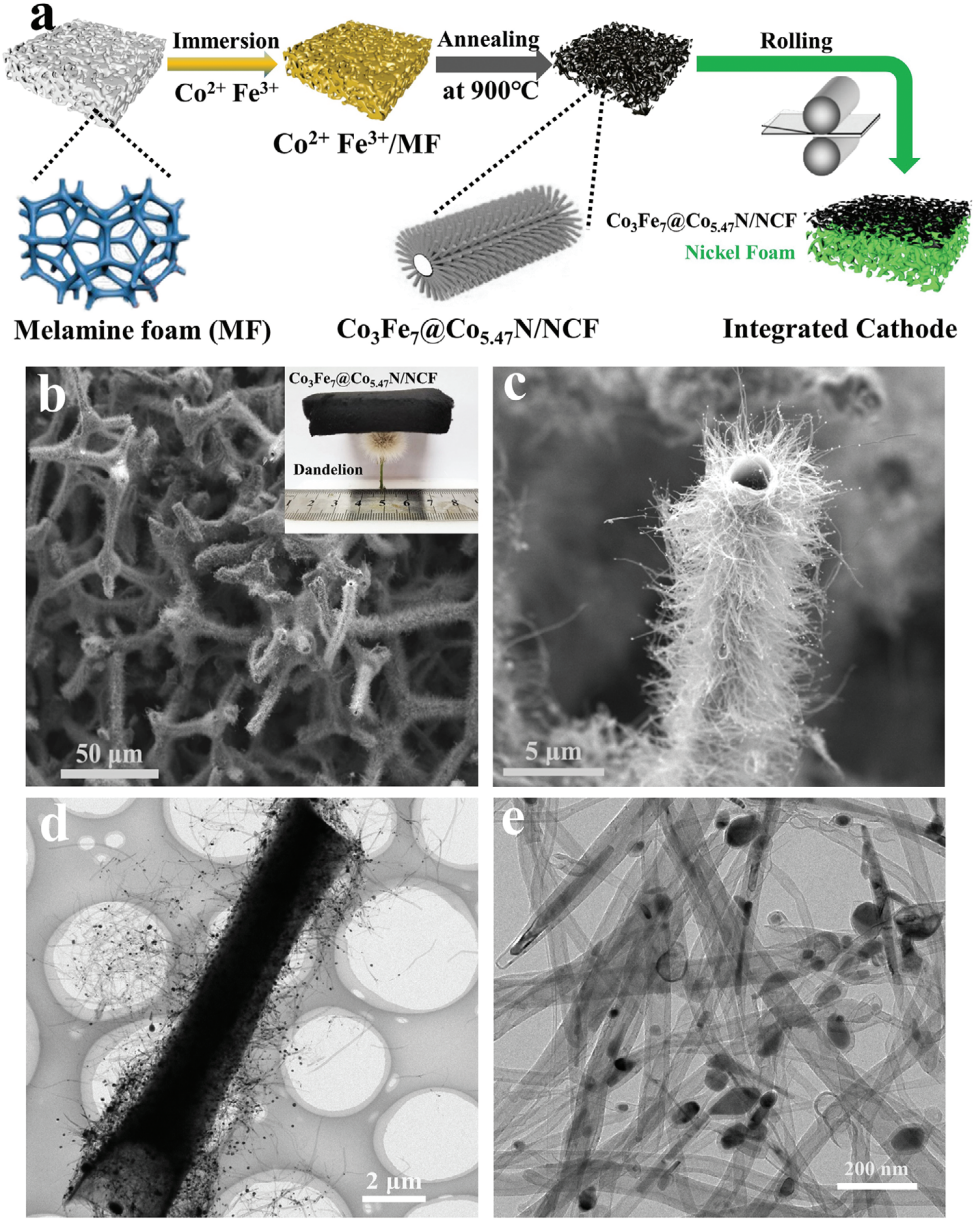
a) Schematic illustration of the synthesis of Co_3_Fe_7_@Co_5.47_N/NCF, b,c) SEM and d,e) TEM images of Co_3_Fe_7_@Co_5.47_N/NCF at different magnifications. The inset image in (b) is a digital image of Co_3_Fe_7_@Co_5.47_N/NCF on a dandelion.

The typical preparation procedure of Co_3_Fe_7_@Co_5.47_N/NCF is schematically illustrated in **Figure** **1**a. The preparation procedure involves a simple impregnation and one‐step pyrolysis. First, the Fe^3+^ and Co^2+^ ions were evenly adsorbed on melamine foam (MF) by a facile immersion method. Then, the melamine foam with iron and cobalt ions was annealed at 900 °C under nitrogen (N_2_) atmosphere for 4 h. During the pyrolysis process, the melamine foam was pyrolyzed to form 3D N‐doped carbon, which inherits the foam structure, and the metal ions were converted to uniform metal nanoalloy and transition metal nitride. Here it is necessary to mention that the ratio of Co to Fe plays an important role in forming the metal nanoalloy and transition metal nitride, which will be discussed later. Meanwhile, the converted CoFe nanoalloys were acted as catalytic sites to generate N‐doped carbon nanotubes in situ grown on the 3D carbon foam. Then, the as‐prepared Co_3_Fe_7_@Co_5.47_N/NCF was directly pressed onto nickel foam current collector to fabricate the integrated air cathode. When adjusting the ratio of Co to Fe, the Co_3_Fe_7_/NCF and Co_5.47_N/NCF were also obtained.

The morphology of the as‐synthesized NCF, Co_5.47_N/NCF, Co_3_Fe_7_/NCF and Co_3_Fe_7_@Co_5.47_N/NCF are displayed in Figure S1 in the Supporting Information and Figure 1b–e. Figure S1a,b in the Supporting Information shows that the formed N‐doped carbon foam displays 3D network structure, and the carbon skeletons are smooth and cross‐linked. When Co precursor was added, the Co_5.47_N/NCF was obtained with a similar morphology of NCF (Figure S1c, Supporting Information). However, the skeleton surface is relatively rough (Figure S1d, Supporting Information). When Fe and Co precursors with a ratio of 0.5:1 were added, the Co_3_Fe_7_@Co_5.47_N/NCF was formed. The inset in Figure 1b shows a digital image of 3D integrated Co_3_Fe_7_@Co_5.47_N/NCF, which is very lightweight and can firmly stand on a dandelion without destroying its fluffs. Figure 1b shows a representative scanning electron microscopy (SEM) image of Co_3_Fe_7_@Co_5.47_N/NCF, which is composed of 3D interconnected and continuous carbon skeletons with a diameter of several micrometers. Interestingly, the carbon skeletons were transformed into hollow structure (Figure 1c), which is explained by the self‐catalytic function of the transition metal alloy in carbon matrix. The hollow structure can expose more active sites, which can facilitate ORR. The transmission electron microscopy (TEM) image in Figure 1d provides details of Co_3_Fe_7_@Co_5.47_N/NCF, demonstrating the hollow carbon skeletons are uniformly covered by a dense layer of carbon nanotubes with a length of 10–15 µm. Moreover, the magnified image shown in Figure 1e shows that the converted metal nanoparticles with a diameter of 10–20 nm are encapsulated in the carbon nanotubes. When more Fe precursor was added (Fe:Co = 2:1), the obtained product is Co_3_Fe_7_/NCF rather than Co_3_Fe_7_@Co_5.47_N/NCF, which is explained that Co is removed from Co_5.47_N to form more CoFe alloy. The obtained Co_3_Fe_7_/NCF displays a similar 3D network structure, however the long carbon skeleton is partially destroyed, and the Co_3_Fe_7_ nanoalloys are grown on the carbon foam (Figure S1e,f, Supporting Information). The results above reveal that the ratio of Fe to Co determines the formation of Co_3_Fe_7_@Co_5.47_N/NCF and maintains the integrity of carbon skeletons.

**Figure 2:**
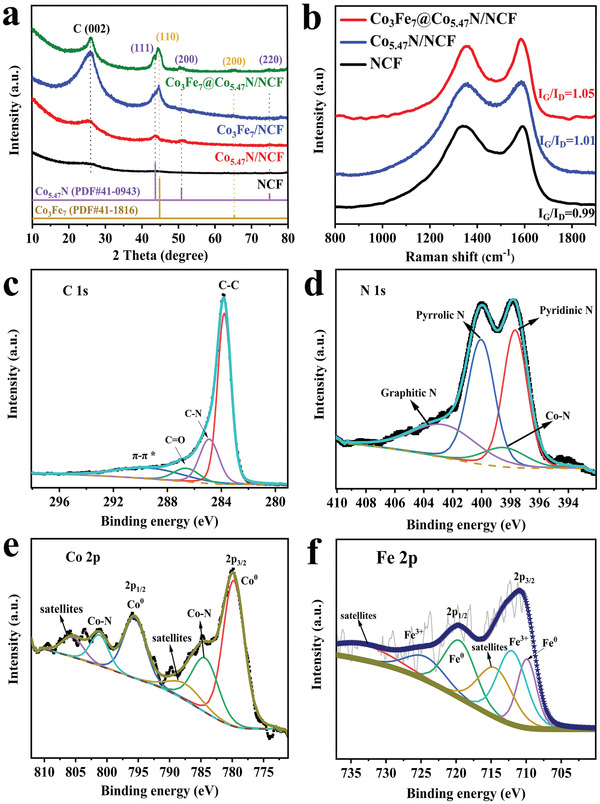
a) XRD patterns and b) Raman spectra of NCF, Co_5.47_N/NCF and Co_3_Fe_7_@Co_5.47_N/NCF. The core‐level c) C 1s, d) N 1s, e) Co 2p, and f) Fe 2p XPS spectra of Co_3_Fe_7_@Co_5.47_N/NCF.

X‐ray diffraction (XRD) patterns of the as‐synthesized NCF, Co_5.47_N/NCF, Co_3_Fe_7_/NCF and Co_3_Fe_7_@Co_5.47_N/NCF are shown in **Figure** **2**a. The wide peak at ≈26° is assigned to the (002) crystal plane of NCF, indicating the formation of graphitic structure during the pyrolysis.^[^ ^24^ ^]^ The obvious diffraction peaks at 44.8° and 65.1° are assigned to (110), (200) crystal planes of Co_3_Fe_7_ alloy (JSPDF No. 48‐1816).^[^ ^25^ ^]^ The diffraction peaks at 43.7°, 50.8°, and 74.9° are assigned to (111), (200) and (220) crystal planes of Co_5.47_N phase (JSPDF No. 41‐0943).^[^ ^2^ ^]^ The XRD patterns strongly indicate the formation of Co_5.47_N and Co_3_Fe_7_ alloy in Co_3_Fe_7_@Co_5.47_N/NCF. Raman spectra in Figure 2b shows D band at ≈1340 cm^−1^ corresponding to disordered carbon and G band at ≈1585 cm^−1^ corresponding to graphite carbon. The value of *I*_G_/*I*_D_ can be used to evaluate the degree of graphitization.^[^ ^26^ ^]^ The *I*_G_/*I*_D_ values of Co_3_Fe_7_@Co_5.47_N/NCF, Co_5.47_N/NCF and NC are 1.05, 1.01, and 0.99 respectively, suggesting that the addition of Fe and Co can enhance the graphitization degree of carbon foam.^[^ ^27^ ^]^ The surface area and pore size distribution of Co_3_Fe_7_@Co_5.47_N/NCF measured by nitrogen adsorption/desorption analysis are shown in Figure S2 in the Supporting Information. The Co_3_Fe_7_@Co_5.47_N/NCF exhibits a type‐IV isotherm with a H_3_‐type hysteresis loop, and the corresponding BET surface area is 786.8 m^2^ g^−1^ with a pore size distribution of 3–4 nm range.^[^ ^28^, ^29^ ^]^ The X‐ray photoelectron spectroscopy (XPS) survey scan of Co_3_Fe_7_@Co_5.47_N/NCF demonstrates the presence of C, N, Fe, and Co (Figure S3, Supporting Information). The deconvoluted high‐resolution C 1s spectrum in Figure 2c displays that the main sharp and strong peaks located at 283.8, 284.9, 286.6, and 291.0 eV are attributed to C—C, C—O, C=N, and *π*—π* bonds, respectively.^[^ ^30^, ^31^ ^]^ The core‐level XPS N 1s spectrum in Figure 2d deconvoluted into four peaks located at 397.7, 398.4, 400.0, and 402.6 eV are assigned to pyridinic N (34.37 at%), N—Co (8.78 at%), pyrrolic N (34.59 at%), and graphitic N (22.26 at%).^[^ ^32^, ^33^ ^]^ The deconvoluted Co 2p spectrum in Figure 2e shows two pairs of peaks. The peaks at 779.90 and 794.70 eV are attributed to Co atom in CoFe alloy, while the peaks located at 784.76 and 797.26 eV correspond to Co—N specie. The peaks at binding energies of 781.6 and 796.4 eV are assigned to the shakeup satellites.^[^ ^34^ ^]^ Similarly, the Fe 2p spectrum is deconvoluted in Figure 2f. The peaks at 709.8 and 719.7 eV are ascribed to the metallic Co_3_Fe_7_ alloy,^[^ ^35^ ^]^ and the other peaks centered at 712.6 and 725.2 eV are attributed to ionic state peaks and the binding energy located at 714.9 and 732.5 eV are shakeup satellite peaks. Electron energy loss spectrum (EELS) was conducted to further confirm the forms of metals in Co_3_Fe_7_@Co_5.47_N/NCF. The representative EELS in Figure S4 in the Supporting Information features the characteristic signals of Fe L_2,3_ and Co L_2,3_ edges at ≈710 and ≈780 eV, respectively.^[^ ^32^, ^36^ ^]^ The line profiles of Fe and Co show two strong L_3_ and L_2_, which are ascribed to the transition of electrons from the spin‐orbit split levels 2p_3/2_ and 2p_1/2_ to unoccupied 3d states. The L_3_ edge of Fe at ≈707.9 eV reveals the form of Fe^0^, which is in good agreement with literature.^[^ ^37^ ^]^ The Co‐L_3_ edge is composed of a fine structure with two features. The peak at ≈779.0 eV is assigned to Co^0^ and the one at ≈781.0 eV is assigned to fully coordinated cobalt (Co—N).^[^ ^38^, ^39^ ^]^ The EELS further evidences the existence of Co—Fe alloy and Co—N at the atomic scale, which is consistent with the observation from XRD and XPS results.

**Figure 3:**
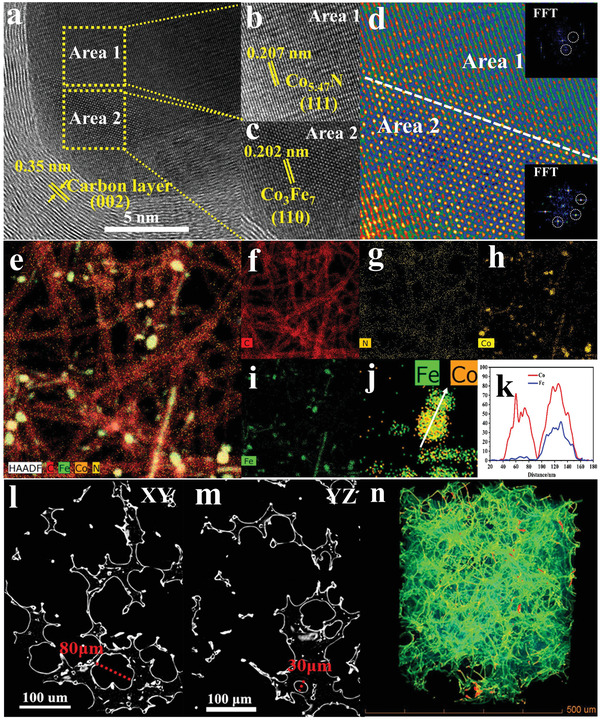
a–c) High‐magnification TEM images of Co_3_Fe_7_@Co_5.47_N/NCF. d) Colored intensity image of the interface structure between Co_3_Fe_7_ and Co_5.47_N. Inset: FFT pattern of Co_3_Fe_7_ and Co_5.47_N. e–j) HAADF–STEM images with elemental mapping of C, N, Co, and Fe. k) The corresponding EDS line‐scan spectrum. l) XY, m) YZ view 2D XRM images, and n) 3D XRM projection of the reconstructed Co_3_Fe_7_@Co_5.47_N/NCF integrated cathode.

The high‐resolution TEM (HRTEM) image of Co_3_Fe_7_@Co_5.47_N/NCF in **Figure** **3**a demonstrates that the lattice spacing of the outer layer is 0.35 nm, corresponding to (002) crystal plane of graphitic carbon, and the Co_5.47_N (Area 1) and Co_3_Fe_7_ nanoalloy (Area 2) are trapped by the carbon layer. Figure 3b,c shows that the lattice fringes with spacing of 0.207 and 0.202 nm are assigned to (111) plane of Co_5.47_N and (110) plane of Co_3_Fe_7_, respectively.^[^ ^40^, ^41^, ^42^ ^]^ Figure 3d presents the interface with distinctly different crystal structure and electron densities between the two phases of Co_3_Fe_7_ and Co_5.47_N in the catalyst. The corresponding fast Fourier transform (FFT) patterns (the inset in Figure 3d) also confirm the structural characteristics, indexed to (110) and (111) in Co_3_Fe_7_ and Co_5.47_N crystal lattices, respectively. Such an interfacial structure is essential to ORR, which will be discussed later. The high angle annular dark‐field scanning transmission electron microscope (HAADF‐STEM) and the corresponding elemental images (Figure 3e–h) identify the elemental distribution in the catalyst, and the corresponding elemental mapping images further reveal the uniform distribution of C, N, Co, and Fe elements within the matrix, indicating homogeneous doping and distribution of active sites in the catalyst. Moreover, the EDS line scan (Figure 3j,k) demonstrates a distinct cross‐over between Co and Fe with significantly different intensities, indicating the presence of the interfacial structure between Co_3_Fe_7_ and Co_5.47_N by Fe modulation. The Co_3_Fe_7_@Co_5.47_N/NCF structure was also panoramically reconstructed and visualized by 3D X‐ray microtomography (XRM). Figure 3l,m shows the axial section from the middle of tomogram, and some super macro‐voids with a size range from 30 to 80 µm are distributed. A significant advantage of 3D X‐ray tomography is the high sensitivity toward material density.^[^ ^43^ ^]^ Co_3_Fe_7_ and Co_5.47_N with high mass densities show much brighter contrast than the surrounding nitrogen doped carbon skeletons. Figure 3n and Video S1 in the Supporting Information show the reconstructed 3D image with a spatial feature of Co_3_Fe_7_@Co_5.47_N/NCF, and the Co_3_Fe_7_@ Co_5.47_N with a high mass density (marked in yellow and red) located on the continuous nitrogen doped 3D carbon branches (marked in green). The result clearly demonstrates the 3D porous structure, which can be functioned as a robust support for Co_3_Fe_7_@Co_5.47_N and ensures efficient penetration of electrolyte and transportation of oxygen into the inner structure.^[^ ^44^, ^45^ ^]^ Therefore, this material can be directly used as an air electrode to realize oxygen reduction reaction with fast mass and charge transport.

**Figure 4:**
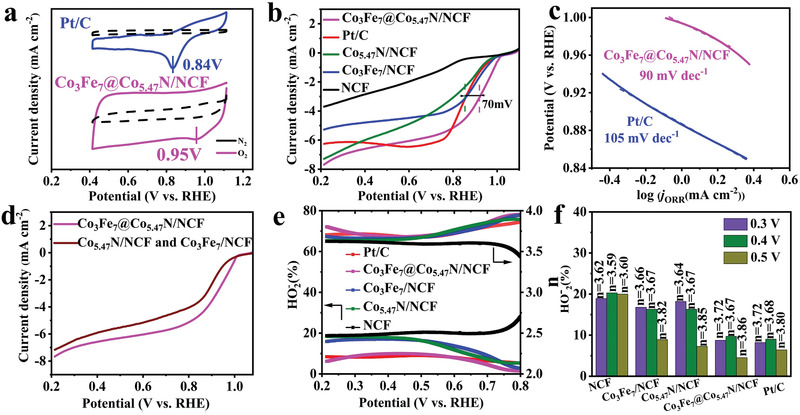
a) CV curves of Co_3_Fe_7_@Co_5.47_N/NCF and Pt/C in O_2_‐/N_2_‐saturated 0.1 m KOH solutions. b) LSV curves of NCF, Co_5.47_N/NCF, Co_3_Fe_7_/NCF, Co_3_Fe_7_@Co_5.47_N/NCF, and Pt/C in O_2_‐saturated 0.1 m KOH solution at a scan rate of 10 mV s^−l^ at 1600 rpm. c) Tafel slope curves. d) LSV curves of Co_3_Fe_7_@Co_5.47_N/NCF and the mixture of Co_5.47_N/NCF and Co_3_Fe_7_/NCF. e,f) Corresponding peroxide yields and electron number.

To profile the relationship between the unique structure and electrocatalytic performance, cyclic voltammetry (CV) and linear sweep voltammetry (LSV) measurements were used to investigate ORR activity of the catalysts. **Figure** **4**a shows the CV curves of Co_3_Fe_7_@Co_5.47_N/NCF catalyst and 20%Pt/C at 5 mV s^−1^ in 0.1 m KOH solution saturated with N_2_ or O_2_. The obvious reduction peak in the O_2_‐saturated solution clearly demonstrates the oxygen reduction on Co_3_Fe_7_@Co_5.47_N/NCF. Moreover, the peak potential on Co_3_Fe_7_@Co_5.47_N/NCF (0.95 V) is more positive than that on Pt/C (0.84 V), indicating a prominent ORR. Additionally, the larger double‐layer current density also indicates a larger electrochemical active area of Co_3_Fe_7_@Co_5.47_N/NCF catalyst. Figure 4b shows the LSV curves of Co_3_Fe_7_@Co_5.47_N/NCF and the reference samples (Co_5.47_N/NCF, Co_3_Fe_7_/NCF, NCF and 20 wt% Pt/C) with RDE measurement at a rotation speed of 1600 rpm. The onset potential on Co_3_Fe_7_@Co_5.47_N/NCF is more positive than those on Co_3_Fe_7_/NCF, Co_5.47_N/NCF or NCF, indicating the synergistic effect of Co_3_Fe_7_ nanoalloy and Co_5.47_N toward ORR. Furthermore, the Co_3_Fe_7_@Co_5.47_N/NCF catalyst exhibits a remarkable onset potential of 1.02 V versus RHE and a half‐wave potential of 0.92 V versus RHE, which are much more positive than those on commercial 20wt% Pt/C (0.98 and 0.85 V, respectively). Figure 4c shows that the slope of Tafel plot is 90 mV dec^−1^, which is lower than that for commercial 20wt% Pt/C (105 mV dec^−1^), indicating the excellent ORR kinetics of Co_3_Fe_7_@Co_5.47_N/NCF. To verify the essential role of the designed interfacial structure in oxygen reduction, Co_3_Fe_7_@Co_5.47_N/NCF was compared with a mixture of Co_5.47_N/NCF and Co_3_Fe_7_/NCF, and the Co_3_Fe_7_@Co_5.47_N/NCF with interfacial structure displays higher catalytic activity toward ORR than the mixture as shown in Figure 4d. The enhanced catalytic activity is attributed to the electronic coupling effect arising from the interface at two phases, which can not only offer active sites but also accelerate electron transfer from the carbon matrix to the catalyst surface,^[^ ^46^ ^]^ and the existence of the interfacial structure benefits electrocatalysis owing to the modulated electronic structure. To gain further insight into ORR kinetics, the K‐L plots extracted from the LSV curves of Co_3_Fe_7_@Co_5.47_N/NCF at rotating speeds ranging from 400 to 1600 rpm are shown in Figure S5 in the Supporting Information. The electron transfer number of Co_3_Fe_7_@Co_5.47_N/NCF calculated from K‐L plots is ≈3.81, demonstrating the efficient four‐electron transfer. To assess the reaction pathways of these catalysts, rotating ring disk electrode (RRDE) measurement was performed in 0.1 m KOH solution at a scan rate of 10 mV s^−1^. Compared with Co_3_Fe_7_/NCF, Co_5.47_N/NCF and NC, the Co_3_Fe_7_@Co_5.47_N/NCF catalyst shows a lower HO_2_^−^ yield of below 10% in the entire potential range (0.2–0.8 V) and a higher electron transfer number of above 3.72 (Figure 4e,f), which is close to or even better than that of the Pt/C catalyst.^[^ ^47^ ^]^ It is instructive to compare the ORR performance of Co_3_Fe_7_@Co_5.47_N/NCF with other similar catalysts from literature, and Table S1 in the Supporting Information lists the onset potentials and half‐wave potentials as well as limiting current densities of various CoFe‐based catalysts in KOH solutions. It's observed that the ORR performance of Co_3_Fe_7_@Co_5.47_N/NCF is superior to many of the previous relevant catalysts.

**Figure 5:**
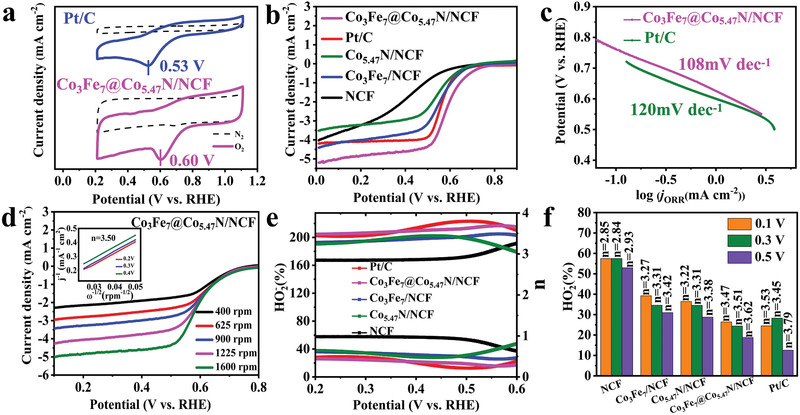
a) CV curves of Co_3_Fe_7_@Co_5.47_N/NCF and Pt/C in O_2_‐saturated and N_2_‐saturated neutral solutions. b) LSV curves of NCF, Co_5.47_N/NCF, Co_5.47_N/NCF, Co_3_Fe_7_@Co_5.47_N/NCF and Pt/C in O_2_‐saturated neutral solution at a scan rate of 10 mV s^−l^ at 1600 rpm. c) Tafel slope curves. d) LSV curves of Co_3_Fe_7_@Co_5.47_N/NCF at various rotation rates and corresponding K‐L plots. e,f) Corresponding peroxide yields and electron number.

Furthermore, the ORR catalytic performance of Co_3_Fe_7_@Co_5.47_N/NCF was also investigated in a neutral solution (3.5 wt% NaCl solution, pH = 7.0) by both RDE and RRDE measurements. As shown in **Figure** **5**a, the Co_3_Fe_7_@Co_5.47_N/NCF shows more positive reduction peak potential (0.60 V) compared with the commercial Pt/C (0.53V), suggesting a prominent ORR activity. Figure 5b shows the LSV curves of the various catalysts. Compared with Co_3_Fe_7_/NCF, Co_5.47_N/NCF and NC, the Co_3_Fe_7_@Co_5.47_N/NCF catalyst exhibits more positive onset potential (0.77 V) and half‐wave potential (0.59 V), which are also more positive than those on the commercial Pt/C (0.72 and 0.57 V respectively). More importantly, the Tafel slope of Co_3_Fe_7_@Co_5.47_N/NCF catalyst (108 mV dec^−1^) is lower than that of the commercial Pt/C catalyst (120 mV dec^−1^), suggesting its superior ORR kinetics (Figure 5c). To gain further insight into ORR kinetics, the LSV curves in Figure 5d suggests that Co_3_Fe_7_@Co_5.47_N/NCF catalyst also undergoes a nearly four‐electron ORR transfer pathway. As shown in Figure 5e,f, the RRDE result displays that the H_2_O_2_ yield on Co_3_Fe_7_@Co_5.47_N/NCF remains below 24% and the average electron transfer is 3.51, which are superior to the commercial Pt/C. It's worth to mention that the kinetics of ORR in the neutral solution is more sluggish, which is mainly due to a low concentration of OH^−^ and the barrier effect of Cl^−^.^[^ ^48^ ^]^

In order to evaluate stability of the catalyst, the accelerated durability tests (ADT) were performed by potential cycling between 0.2 and 1.0 V versus RHE in both alkaline and neutral solutions for 5000 cycles at a scan rate of 100 mV s^−1^. Figure S6a,b in the Supporting Information shows that there is no obvious shift in the half‐wave potential after 5000 cycles in both alkaline and neutral solutions, indicating the high stability and durability of Co_3_Fe_7_@Co_5.47_N/NCF. In order to further analyze stability of the catalyst, HRTEM image of Co_3_Fe_7_@Co_5.47_N/NCF after 5000 cycles is shown in Figure S7 in the Supporting Information. Obviously, the interfacial structure between the two phases of Co_3_Fe_7_@Co_5.47_N/NCF catalyst still exists, implying that the catalyst is very stable to provide rich active sites during ORR process. To further understand the roles of Co_3_Fe_7_ and Co_5.47_N in electrocatalytic performance, the Co_3_Fe_7_@Co_5.47_N/NCF was etched in an acid solution to partially remove the metal alloy and metal nitride, and the etched sample displays a significant decay in electrocatalytic activity in either alkaline or neutral solution (Figure S8, Supporting Information), further confirming the Co_3_Fe_7_ alloy and Co_5.47_N play a vital role in promoting the electrocatalytic activity toward ORR. The electrochemical surface areas (ECSA) of these materials estimated from the double‐layer capacitance (*C*_dl_) are 16.95, 12.97, 6.82, and 1.14 mF for Co_3_Fe_7_@Co_5.47_N/NCF, Co_3_Fe_7_/NCF, Co_5.47_N/NCF, and NCF, respectively. Additionally, the ECSA of Co_3_Fe_7_@Co_5.47_N/NCF is 15 folds of that for NCF (Figures S9 and S10, Supporting Information), suggesting the active sites are fully exposed and highly available due to the in situ transformation.

**Figure 6:**
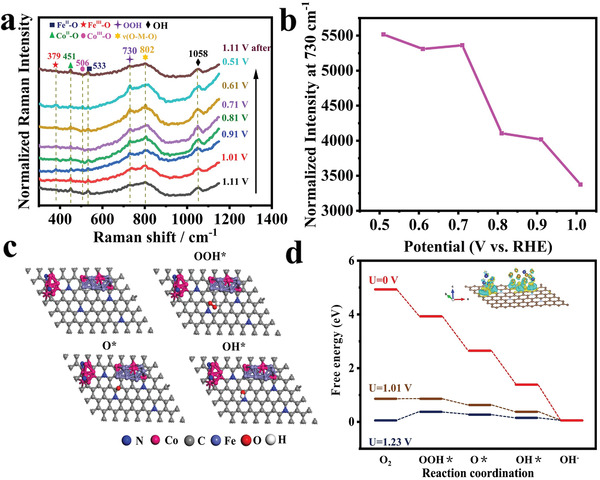
a) Potential‐dependent in situ Raman spectra during ORR process. b) Normalized Raman intensity of the stretching mode of OOH at 730 cm^−1^. c) Optimized structures of OH*, O*, and OOH* intermediates on Co_3_Fe_7_@Co_5.47_N/NCF. d) Free energy diagram of Co_3_Fe_7_@Co_5.47_N/NCF at zero potential (*U*  = 0), equilibrium potential (*U*  = 1.23 V), and thermodynamic limiting potential.

In situ electrochemistry Raman spectra with an applied potential range from 1.11 to 0.51 V on Co_3_Fe_7_@Co_5.47_N/NCF catalyst using a home‐made electrochemical cell (Figure S11, Supporting Information) for ORR were recorded. **Figure** **6**a displays the Raman spectra of Co_3_Fe_7_@Co_5.47_N/NCF polarized at different potentials. At an initial potential of 1.11 V versus RHE, the oxygen reduction reaction does not occur, and two bands appearing at 451 and 533 cm^−1^ are attributed to Co^II^—OH and Fe^II^—OH, respectively.^[^ ^49^, ^50^ ^]^ Shifting the potential to the onset potential of 1.01 V (where ORR occurs), three new bands appear at 379, 506, and 730 cm^−1^, which are assigned to *ν*(Fe^III^—O), *ν*(Co^III^—O) vibrations and O—O stretching of OOH, respectively.^[^ ^51^, ^52^ ^]^ When further negatively shifting the potential, the intensities of these bands increase and the bands for Co^II^—O and Fe^II^—O gradually disappear, which are due to the conversion of Co^II^—O/ Fe^II^—O to Co^III^—O/ Fe^III^—O species during ORR. In addition, the band observed at 802 cm^−1^ is the characteristic of *ν*(O—M—O) vibration in both Co—O—Co and Fe‐O‐Fe species, implying the metal‐oxygen unites as the main ORR active sites.^[^ ^53^, ^54^ ^]^ The band observed at 1058 cm^−1^ is assigned to OH deformation mode due to the existence of alkaline solution and the generation of OH^−^. When the potential shifted back from 0.51 to 1.11 V, the Raman bands for Co^II^—O/ Fe^II^—O reappear and the other bands (Co^III^—O/ Fe^III^—O) gradually disappear. Figure 6b shows the normalized Raman intensity of band at 730 cm^−1^, which is ascribed to the O—O stretching of OOH during ORR process.^[^ ^55^ ^]^ The intensity increases when shifting the potential from 1.11 to 0.51 V, suggesting much more accumulation of oxygen intermediates. Therefore, the ORR mechanism is proposed that the OH species are first adsorbed on the main catalytic sites to form Co^II^—OH/ Fe^II^—OH and then Co^II^—OH/ Fe^II^—OH species interacting with O_2_ to generate Co^III^—OH/ Fe^III^—OH and OOH* as a key intermediate. The findings can give a deep understanding of the elementary processes and provide evidence for density functional theory (DFT) simulation.

DFT simulations via Vienna Ab initio simulation package (VASP) were performed to understand the role of Co_3_Fe_7_@Co_5.47_N/NCF in ORR, and details are given in Supporting Information. The optimal structures of NCF, Co_5.47_N/NCF and Co_3_Fe_7_@Co_5.47_N/NCF are shown in Figure 6c and Figures S12–S15 (Supporting Information) to investigate the favorable active sites and free energy pathways, and the calculated binding free energies of the reaction intermediates are shown in Table S2‐S4. For NCF, the binding free energies of OOH*, O* and OH* intermediates are 4.17, 2.62, and 1.27 eV. For Co_5.47_N/NCF, the binding free energies of OOH*, O* and OH* are 4.09, 2.59, and 1.30 eV. The binding free energies of OOH*, O*, and OH* intermediates on Co_3_Fe_7_@Co_5.47_N/NCF are 3.91, 2.55, and 1.25 eV, which are close to that of an ideal catalyst. Figure 6d shows the calculated free energy pathways of four‐electron ORR reaction at zero potential, equilibrium potential and limiting potential. Clearly, all elementary reactions can occur spontaneously at *U* = 0 V (zero potential) and the free energy pathway is downhill. At the equilibrium potential (*U* = 1.23 V), the elementary reactions along the four‐electron pathway are endergonic and thus thermodynamically unfavorable. The limiting potential *U*_limiting_ (maximum external potential) at which all the ORR elementary steps on Co_3_Fe_7_@Co_5.47_N/NCF are still exothermic is 1.01 V, which is much more positive than the calculated *U*_limiting_ for NCF (0.75 V) or Co_5.47_N/NCF (0.83V), confirming the superior catalytic activity of Co_3_Fe_7_@Co_5.47_N/NCF toward ORR. The DFT analysis fully supports the experimental results, stressing the key role of interfacial structure between Co_3_Fe_7_ and Co_5.47_N in promoting ORR.

**Figure 7:**
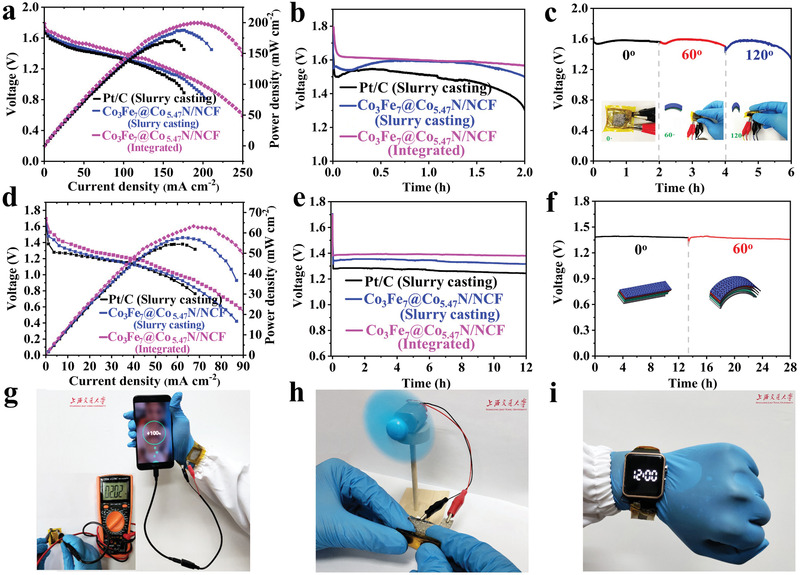
a) Discharge polarization plots and the corresponding power curves of alkaline flexible Al‐air batteries with Co_3_Fe_7_@Co_5.47_N/NCF integrated cathode, Co_3_Fe_7_@Co_5.47_N/NCF slurry casted cathode and 20% Pt/C cathode. b) Galvanostatic discharge curves at a current density of 30 mA cm^−2^. c) Discharge curves of the alkaline flexible Al‐air battery at different bending angles. d) Polarization plots and the corresponding power densities and e) Discharge curves at a current density of 5 mA cm^−2^ of the neutral flexible Al‐air batteries with various cathodes. f) Galvanostatic discharge curves at different bending angles. Demonstration of g) the alkaline flexible Al‐air batteries in four series charging a mobile phone, h) the bent Al‐air battery in neutral gel electrolyte powering a mini fan and i) neutral flexible Al‐air batteries connected in series to power a watch at bending state.

The discharge performance and flexibility of Al‐air batteries with the integrated and additive‐free air cathodes based on Co_3_Fe_7_@Co_5.47_N/NCF were investigated. Meanwhile, air cathodes based on Co_3_Fe_7_@Co_5.47_N/NCF were also prepared through traditional slurry‐casting for comparison to demonstrate the advantage of integrated cathodes. The different preparation processes of integrated and slurry casting air cathodes are described in Figure S16 in the Supporting Information. Generally, slurry casting of air cathode requires sophisticated apparatus and procedures, especially non‐conductive binder is necessary. Additionally, the aggregation of nano‐catalysts is unavoidable. The alkaline flexible Al‐air batteries were fabricated by sandwiching the air cathode, polymer electrolyte and Al‐Mg‐Sn foil anode, and each component is flexible, as illustrated in Figure S17 in the Supporting Information. **Figure** **7**a shows the representative polarization plots and power density curves of Al‐air batteries with integrated cathode and slurry casting cathode as well as commercial Pt/C cathode in an alkaline gel electrolyte. The Al‐air battery with Co_3_Fe_7_@Co_5.47_N/NCF integrated cathode displays a maximum power density of 199.6 mW cm^−2^, which is larger than that with Co_3_Fe_7_@Co_5.47_N/NCF slurry casting cathode (187.7 mW cm^−2^) or Pt/C slurry casting cathode (171.3 mW cm^−2^). Figure 7b shows the discharge curves of the three Al‐air batteries with different cathodes at a current density of 30 mA cm^−2^. The flexible Al‐air battery with Co_3_Fe_7_@Co_5.47_N/NCF integrated cathode maintains a stable discharge plateau of 1.61V, which is larger than those with Co_3_Fe_7_@Co_5.47_N/NCF slurry casting cathode and Pt/C cathode, further demonstrating high performance of the integrated cathode. The discharge capacities of various alkaline Al‐air batteries normalized to mass of the consumed Al alloy are revealed in Figure S18a in the Supporting Information. The Al‐air battery with Co_3_Fe_7_@Co_5.47_N/NCF integrated air cathode delivers a capacity of 1997.3 mAh g^−1^, which is larger than those with Co_3_Fe_7_@Co_5.47_N/NCF slurry casted cathode (1911.3 mAh g^−1^) and Pt/C cathode (1773.9 mAh g^−1^). Figure S18b in the Supporting Information shows rate performance of the alkaline Al‐air battery with Co_3_Fe_7_@Co_5.47_N/NCF integrated cathode. The Al‐air battery displays working voltage plateaus of 1.58, 1.49, 1.39, 1.34, and 1.20 V at the current densities of 30, 60, 90, 120, 150 mA cm^−2^, respectively, and the working voltage can be recovered when switching back the current density to 30 mA cm^−2^. Figure 7c shows the discharge curves of the alkaline flexible Al‐air battery at different bending angles, and there is nearly no voltage decay, demonstrating good flexibility. Additionally, Al‐air battery can also be mechanically recharged for continuous power supply by replacing Al anode or electrolyte. To exam charge capability, the discharge performance of Al‐air battery using the recycled gel electrolyte and a new Al anode was studied, and the Al‐air battery after charging displays no difference in discharge voltage (Figure S19, Supporting Information). Although Al‐air battery is a primary cell, it can be charged by replacing Al alloy anode. The Al anode is oxidized and consumed during discharge, and the discharge stops when Al anode is completely consumed. When the cell is re‐filled with a new Al anode, it can discharge again, and this process is called mechanical recharge.

Neutral electrolytes are more environmentally friendly for wearable electronics, and it is of great interests to explore flexible Al‐air batteries with neutral electrolytes. The maximum power density of the Al‐air battery with Co_3_Fe_7_@Co_5.47_N/NCF integrated cathode is 65.0 mW cm^−2^, which is larger than that with Co_3_Fe_7_@Co_5.47_N/NCF slurry casting cathode or with commercial Pt/C cathode (Figure 7d). Figure 7e displays the discharge curves of the flexible Al‐air batteries with three different cathodes at a current density of 5 mA cm^−2^. The flexible Al‐air battery with Co_3_Fe_7_@Co_5.47_N/NCF integrated cathode delivers a stable working voltage of ≈1.40 V, which is larger than 1.31 V for that with Co_3_Fe_7_@Co_5.47_N/NCF slurry casting cathode and 1.27 V for that with 20% Pt/C. Similar to the phenomenon in the alkaline electrolyte, the neutral Al‐air battery with Co_3_Fe_7_@Co_5.47_N/NCF integrated cathode delivers the maximum specific capacity of 2761 mAh g^−1^ at a current density of 2 mA cm^−2^, which outperforms the neutral Al‐air battery with Co_3_Fe_7_@Co_5.47_N/NCF slurry casted cathode or Pt/C cathode (Figure S20a, Supporting Information). Figure S20b in the Supporting Information shows the discharge rate performance of neutral Al‐air battery, and a high working voltage of 1.15 V is delivered at a large current density of 60 mA cm^−2^, revealing a good rate performance. The results further confirm the electrocatalyst and configuration of air cathode can effectively improve Al‐air battery performance. When the Al‐air battery is bent, there is no performance decay, demonstrating good flexibility (Figure 7f).

The discharge performance of this flexible Al‐air battery is also compared with other flexible Al‐air Batteries reported in literature. Table S5 in the Supporting Information lists the maximum power density and the corresponding discharge voltage of various Al‐air batteries, clearly the flexible Al‐air battery in this work displays the largest power density, demonstrating the superiority of the unique configuration of the air cathode. To demonstrate practical application, one alkaline flexible Al–air battery displays an open circuit voltage of 2.02 V, and four alkaline flexible Al–air batteries connected in series under bending can effectively charge a mobile phone (Figure 7g). Figure 7h demonstrates a bent neutral Al‐air battery can power a fan, implying good flexibility. Figure S21 in the Supporting Information demonstrates four neutral Al‐air batteries in series display a high open‐circuit voltage of 7.73 V. Additionally, the bent neutral Al‐air batteries were fixed in a watch strap to power a smart watch (Figure 7i). These results reveal that a promising potential of Al‐air battery with Co_3_Fe_7_@Co_5.47_N/NCF cathode for application in flexible and wearable electronics.^[^ ^56^, ^57^ ^]^

## 3 Conclusions

In summary, we have developed an additive‐free and integrated air cathode based on highly efficient 3D Co_3_Fe_7_@Co_5.47_N/NCF electrocatalyst with interfacial structure for flexible Al‐air batteries. Benefiting from the unique interfacial structure as well as the highly intrinsic activity of Co_5.47_N and Co_3_Fe_7_ encapsulated in 3D N‐doped carbon foam, the as‐prepared Co_3_Fe_7_@Co_5.47_N/NCF catalyst outperforms the commercial Pt/C toward ORR in both neutral and alkaline electrolytes. Moreover, the ORR process on Co_3_Fe_7_@Co_5.47_N/NCF has been uncovered by in situ electrochemical Raman spectroscopy, confirming the main ORR intermediates are OOH*, M(III)‐OH. The superior performance is also supported by DFT calculation. The fabricated additive‐free and integrated cathode shows better discharge performance than the slurry casting cathode based on the same electrocatalyst. The fabricated Al‐air batteries display good flexibility and deliver a maximum power density of 199.6 mW cm^−2^ in NaOH electrolyte and 65.0 mW cm^−2^ in NaCl electrolyte. This work presents a new avenue to explore integrated electrodes for flexible metal‐air batteries to meet the demand of smart electronic devices.

## 4 Experimental Section

### Materials Synthesis

0.4 g of melamine foam (Kexin Trade Co. Ltd., China) was soaked in 100 mL of 1 × 10^−3^ m FeCl_3_ (≥ 99.9%, Sigma‐Aldrich) and 2 × 10^−3^ m C_4_H_6_O_4_Co (≥ 99.9%, Sigma‐Aldrich) mixed solution for 12 h to adsorb Fe^3+^ and Co^2+^, and then the melamine foam with Fe^3+^ and Co^2+^ was dried at 80 °C overnight. The obtained melamine foam with Fe^3+^ and Co^2+^ was then heated to 900 °C at a ramp rate of 5 °C min^−1^ and maintained for 4 h in a tube furnace under nitrogen flow. The Co_3_Fe_7_/NCF and Co_5.47_N/NCF were also synthesized with the identical procedure by carefully adjusting the ratio of Fe to Co of 2:1 and 0:1, respectively. Nitrogen‐doped carbon foam (NCF) was also synthesized for comparison by annealing the melamine foam at the same temperature.

### Materials Characterization

X‐ray diffraction (XRD, Ultima IV, Rigaku) was used to examine the crystal phase of catalysts with Cu k*α* radiation (*λ* = 0.15 418 nm) at a scanning speed of 2^o^ min^−1^. Morphology and structure of the obtained samples were obtained by Field‐emission scanning electron microscope (FE‐SEM, MIRA3 LHM, TESCAN) and transmission electron microscopy (FE‐TEM, TALOS F200X, FEI) with an accelerating voltage of 5 and 200 kV, respectively. X‐ray photoelectron spectroscopy (XPS, AXIS UltraDLD, Kratos) was conducted with Al K*α* X‐ray source. Raman spectra were collected on an inVia Qontor confocal Raman microscope (Renishaw) using a 532 nm laser. X‐ray microscopy (XRM) measurement was carried out using a Zeiss Xradia 520 Versa with a microfocus tube with 50 kV acceleration voltage.

### Electrochemical Measurements

Electrochemical performance of the as‐prepared catalysts was evaluated in both alkaline and neutral solutions with a three‐electrode configuration on Gamry REF 600. Glassy carbon (GC, 5 mm in diameter, PEEK, PINE Instrument Inc.) modified electrode as working electrode, graphite electrode as counter electrode and saturated calomel electrode (SCE) as reference electrode. Before use, GC electrode was polished with alumina polishing powder (0.05 µm) and sonicated in deionized water and ethanol. Catalyst inks were prepared by dispersing 4 mg of as‐synthesized catalyst (Co_3_Fe_7_@Co_5.47_N/NCF, Co_3_Fe_7_/NCF, Co_5.47_N/NCF, NCF or 20wt% Pt/C) in 1 mL of solution (240 µL of ethanol, 720 µL of water and 40 µL of 5% Nafion) and sonicated for 1 h to obtain a uniform dispersion ink (4 mg mL^−1^). Then, 20 µL of the catalyst dispersion was deposited on the surface of GC electrode and dried at room temperature, and the loading mass of catalyst was 0.40 mg cm^−2^. ORR performance of the catalysts was evaluated using cyclic voltammetry (CV) and linear sweep voltammetry (LSV) techniques in 0.1 m KOH (≥ 99.9%, Sigma‐Aldrich) solution or 3.5 wt% NaCl (≥99.9%, Sigma‐Aldrich) solution. Prior to electrochemical testing, electrolytes were saturated with oxygen or nitrogen. LSV curves were recorded with a potential window ranging from 0.1 to −0.8 V (vs SCE in alkaline solution) and 0.455 to −0.644 V (vs SCE in neutral solution) at a scan rate of 10 mV s^−1^. All potentials in this work were converted to the reversible hydrogen electrode (RHE) by the formula: *E*_RHE_ = *E*_SCE_ + 0.059pH+ 0.241. RRDE measurement was also conducted on a RRDE configuration with GC disk and polycrystalline Pt. All the obtained curves were corrected by IR‐compensation. The following equations were used to calculate the number of electron transfer (*n*) and hydrogen peroxide yield

(1) $$\%\left(H_{2}O_{2}\right)=200\times \frac{I_{r}/N}{I_{d}+I_{r}/N}$$

(2) $$n=4\times \frac{I_{d}}{I_{d}+I_{r}/N}$$

where *I*_r_ and *I*_d_ are the ring and disk current densities, and *N* is the ring current collection efficiency (0.34).

### In Situ Electrochemical Raman Spectroscopy

In situ electrochemical Raman measurement was carried out with a three‐electrode configuration using the catalyst modified GC electrode as working electrode, a Pt wire as counter electrode and the SCE reference electrode. Raman spectra were recorded with a Renishaw inVia Qontor confocal Raman microscope (50X objective) using 532 nm laser light (a laser power of 10 mW). The sample was applied to various potentials, and the Raman spectra were collected for 120 s for each test.

### Calculations Details

DFT calculations were performed in the Vienna ab initio simulation package (VASP).^[^ ^58^, ^59^ ^]^ A spin‐polarized GGA PBE functional, all‐electron plane‐wave basis sets with an energy cutoff of 400 eV, and a projector augmented wave (PAW) method were adopted. Co_3_Fe_7_ was simulated using a surface model of p (1 × 1) unit cell periodicity. Co_5.47_N was simulated using a surface model of p (1 × 1) unit cell periodicity. A (3 × 3 × 1) Monkhorst‐Pack mesh was used for the Brillouin‐zone integrations to be sampled. 15 Å vacuum layer was added to avoid the interaction between adjacent layers. The conjugate gradient algorithm was used in the optimization. The convergence threshold was set 1*10^−4^ eV in total energy and 0.05 eV Å^−1^ in force on each atom.^[^ ^60^ ^]^

### Flexible Al‐Air Battery Testing

Flexible Al‐air batteries were fabricated by sandwiching the prepared integrated air cathode, gel electrolyte and Al‐Mg‐Sn alloy foil. The Al‐Mg‐Sn alloy was casted using high purity aluminum, magnesium particles and tin particles (99.99%) in a resistance furnace at a temperature of 760 ± 5 °C, and the nominal composition was Al‐1.0wt% Mg‐0.1wt% Sn. The Co_3_Fe_7_@Co_5.47_N/NCF was directly pressed onto Ni foam as the air electrode. The gel electrolyte was prepared as follows: polyvinyl alcohol (PVA) powder (1.0 g) was dissolved in water (10 mL) at 90 °C under magnetic stirring until the solution became uniform. Then, 1.0 mL of 18.0 m KOH aqueous solution containing 0.02 m zinc acetate was added into the gel. After that, the gel was poured into a PTFE mold to form a thin film and kept in refrigerator at −20 °C over 12 h to cross‐link the electrolyte, and the gel was thawed at room temperature. Thearea of air cathode was 4 cm^2^. The flexible Al‐air batteries were tested with LAND testing system (LAND Electronics Ltd.) at room temperature. Galvanostatic discharge and polarization measurements were used to evaluate discharge performance of flexible Al‐air batteries.

## Conflict of Interest

The authors declare no conflict of interest.

## Supporting information

Supporting Information

Supplemental Movie 1
