# Supplementary material for: Integrated and Binder‐Free Air Cathodes of Co3Fe7 Nanoalloy and Co5.47N Encapsulated in Nitrogen‐Doped Carbon Foam with Superior Oxygen Reduction Activity in Flexible Aluminum‐Air Batteries
Source: Adv Sci (Weinh). 2020 Aug 5;7(18):2000747. doi: 10.1002/advs.202000747 (PMC7509645; doi:10.1002/advs.202000747)
Supplement: Supplementary file 1 — Supporting Information [file ADVS-7-2000747-s001.pdf]

2020 Wiley-VCH GmbH

ADVANCED SCIENCE
Open Access

Supporting Information

for *Adv. Sci.*, DOI: 10.1002/advs.202000747

Integrated and Binder-Free Air Cathodes of Co_3Fe_7 Nanoalloy and $Co_{5.47}N$ Encapsulated in Nitrogen-Doped Carbon Foam with Superior Oxygen Reduction Activity in Flexible Aluminum-Air Batteries

Min Jiang, Chaopeng Fu, Ruiqi Cheng, Wei Zhang, Tongyao Liu, Ruibin Wang, Jiao Zhang, and Baode Sun*

Supporting Information

Integrated and Binder-free Air Cathodes of Co_3Fe_7 Nanoalloy and $Co_{5.47}N$ Encapsulated in Nitrogen-doped Carbon Foam with Superior Oxygen Reduction Activity in Flexible Aluminum-air Batteries

Min Jiang,[1] Chaopeng Fu,[1,*] Ruiqi Cheng,[1] Wei Zhang,[2] Tongyao Liu,[1] Ruibin Wang,[3] Jiao Zhang,[1] Baode Sun[1]

1 School of Materials Science and Engineering, Shanghai Jiao Tong University, Shanghai 200240, P. R. China

2 Advanced Technology Institute, University of Surrey, Guildford, GU2 7XH, UK.

3 Instrumental Analysis Center of SJTU, Shanghai Jiao Tong University, Shanghai 200240, P. R. China

Corresponding Author

chaopengfu@sjtu.edu.cn (Chaopeng Fu)

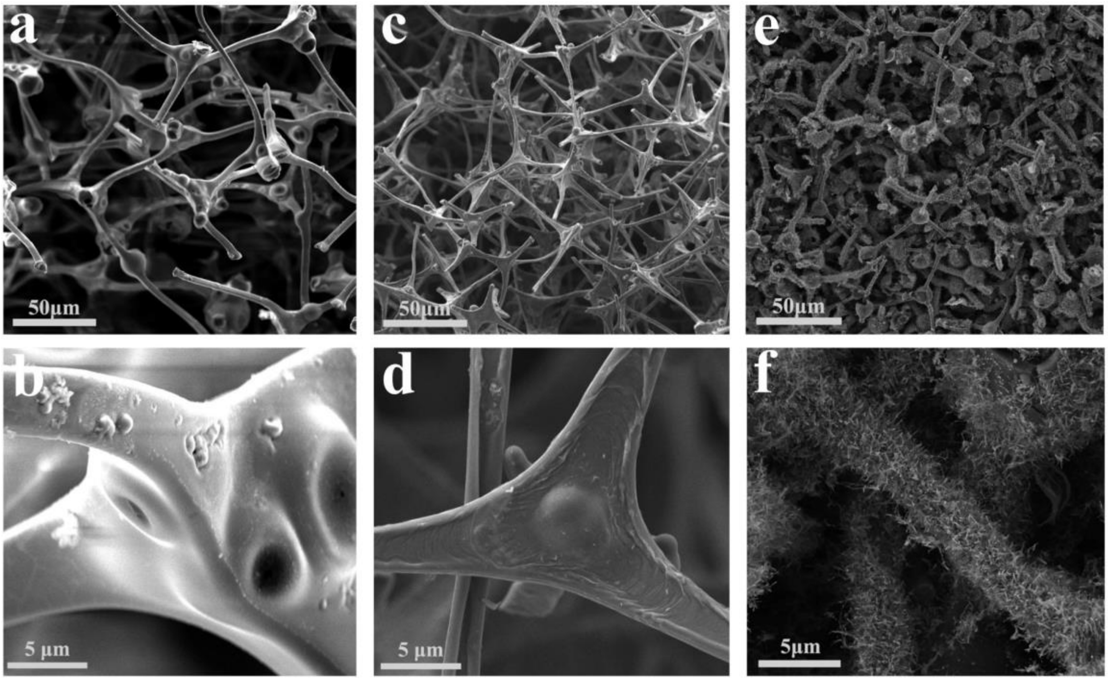

Figure S1. Typical SEM images of (a, b) NCF, (c, d) $Co_{5.47}N$/NCF and (e, f) Co_3Fe_7/NCF.

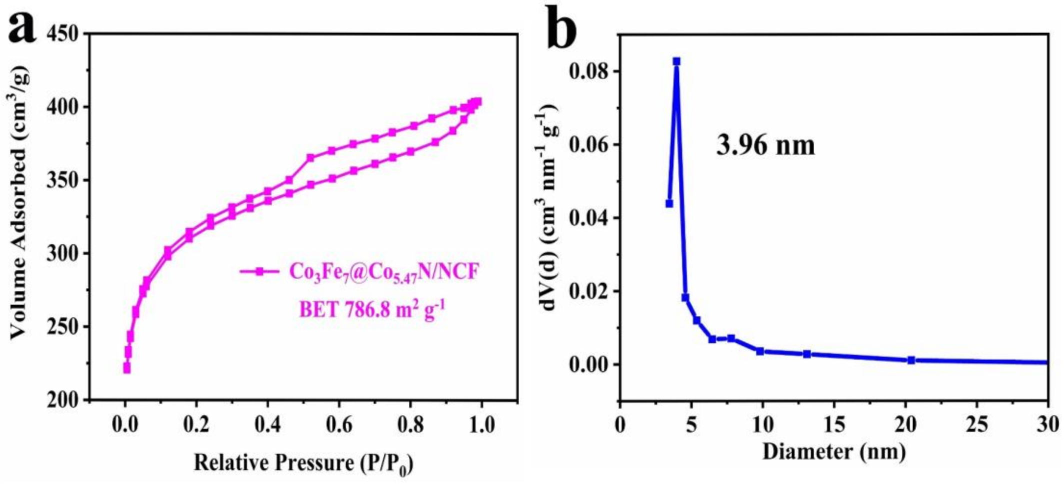

Figure S2. (a) N_2 adsorption/desorption isotherms and (b) pore size distribution of $Co_3Fe_7@Co_{5.47}N/NCF$.

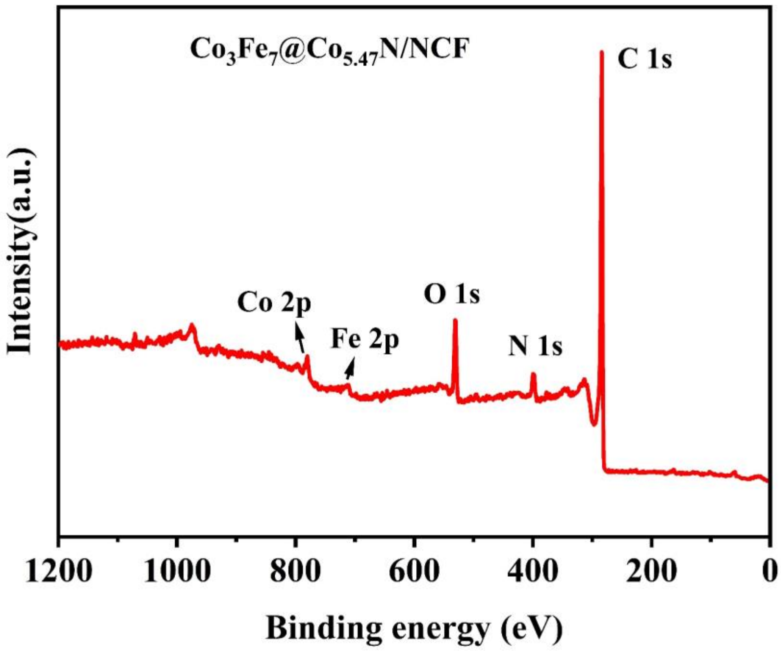

Figure S3. Long-range XPS spectrum of $Co_3Fe_7@Co_{5.47}N/NCF$.

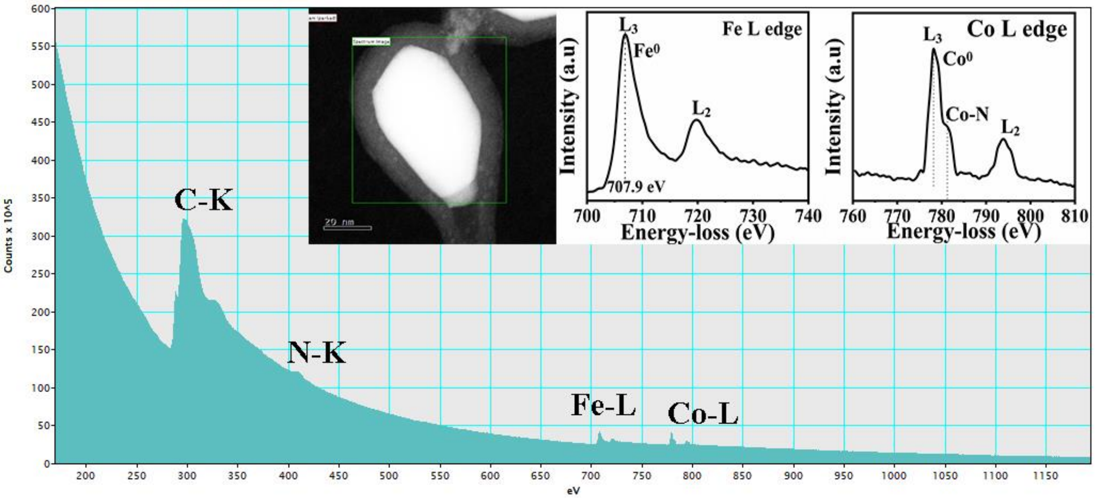

Figure S4. EELS of $Co_3Fe_7@Co_{5.47}N$/NCF.

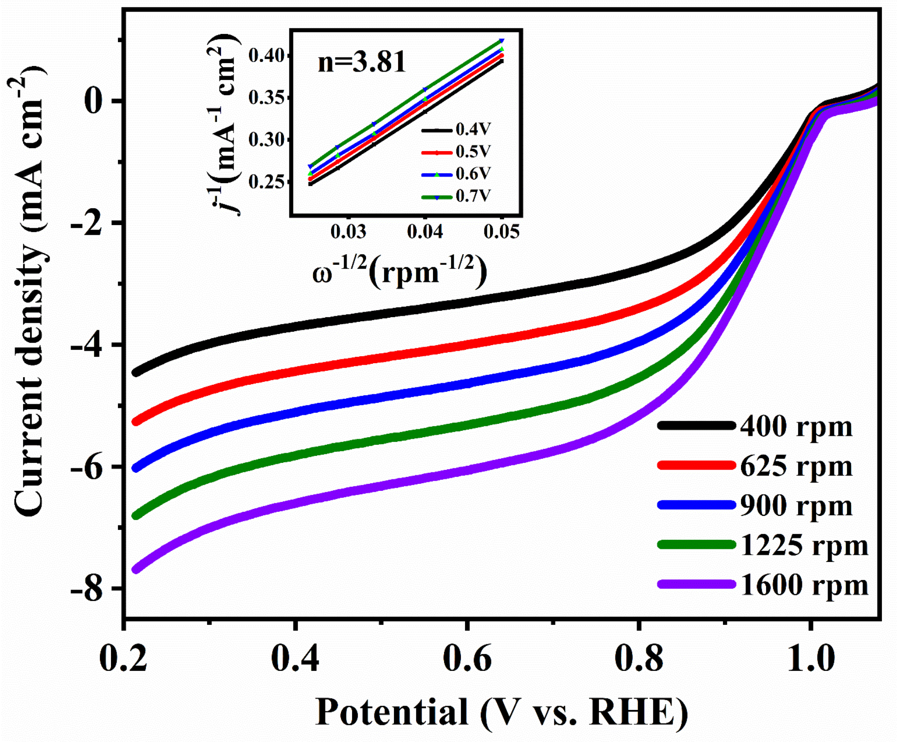

Figure S5. LSV curves of Co_3Fe_7@$Co_{5.47}N$/NCF at various rotation rates and corresponding K-L plots in O_2-saturated alkaline solution.

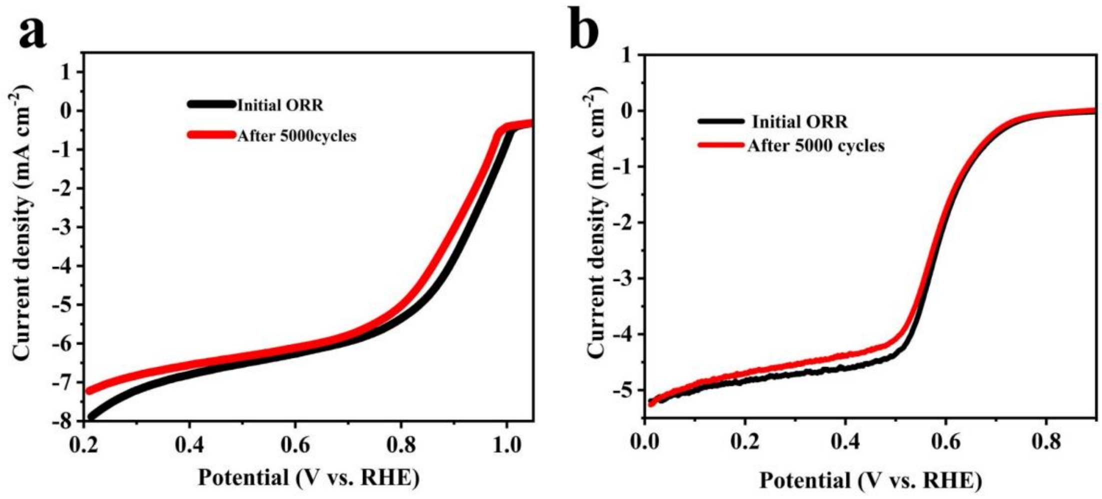

Figure S6. LSV curves of Co_3Fe_7@$Co_{5.47}N$/NCF before and after 5000 CV cycles at a rotation speed of 1600 rpm in O_2 saturated (a) alkaline and (b) neutral solution.

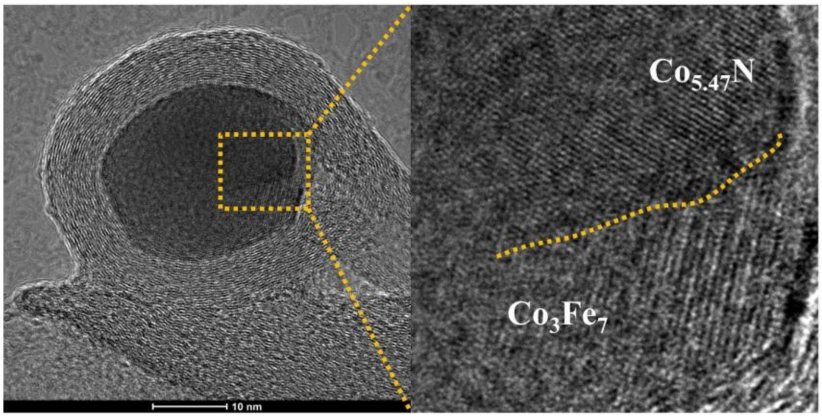

Figure S7. HRTEM images of Co_3Fe_7@$Co_{5.47}N$/NCF after 5000 cycles at different magnifications.

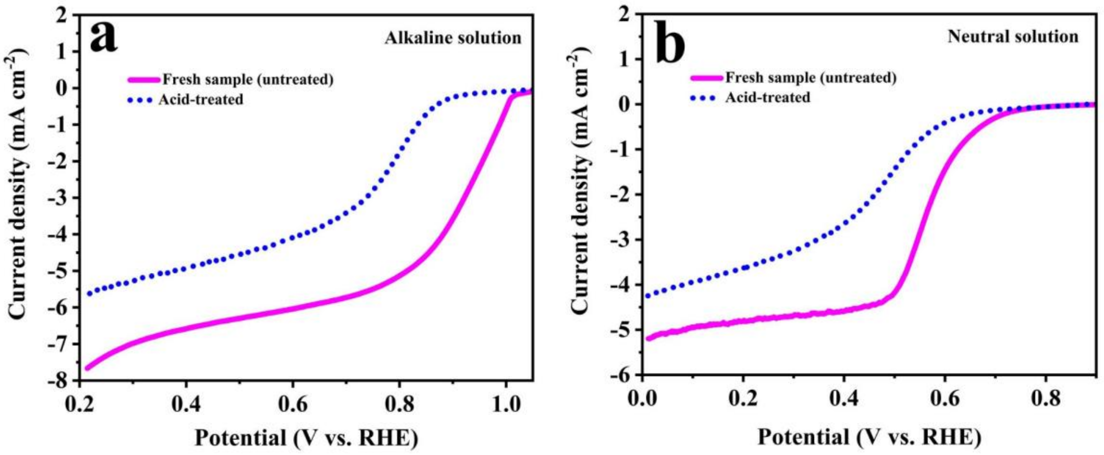

Figure S8. LSV curves of $Co_3Fe_7@Co_{5.47}N$/NCF after acid etching in O_2 saturated (a) alkaline and (b) neutral solution.

1. The electrochemical active surface area (ECSA)

The electrochemical active surface area (ECSA) was determined by the double-layer capacitance of the as-prepared catalyst in the range of -0.02 to 0.08 V vs. SCE. CV measurement was conducted at various scan rates (2, 4, 6, 8, 10, 12 mV s^{-1}). The corresponding relationship between the current from the cyclic voltammogram (i_c) and the scan rate (ν) was given in

$$i_c = v \times C_{DL}$$

To uncover the active role of $Co_{5.47}N$ and Co_3Fe_7, the electrochemical active surface area (ECSA) was calculated from the double-layer capacitance (C_{dl}) [1, 2]. A series of CV curves with potentials in the range of 0.99 to 1.09 V (vs. RHE) were measured, as shown in Figure S9 and S10a.

The C_{dl} was calculated by plotting the capacitive current at 1.04 V (vs. RHE) against the scan rate and fitting with a linear fit (Figure S10b). The results show that there is a linear relationship between ECSA and C_{dl}, where C_{dl} is half of the linear fitting slope[3]. Therefore, the C_{dl} can be employed to describe the ECSA's trends.

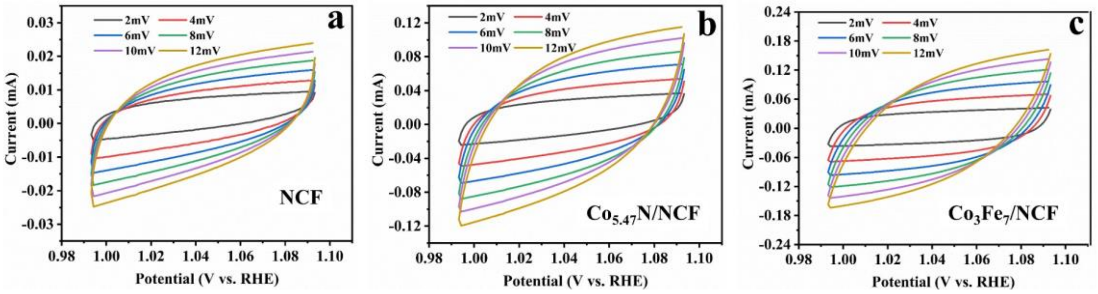

Figure S9. (a-c) Capacitive CV curves of NCF, $Co_{5.47}N$/NCF and Co_3Fe_7/NCF, respectively, recorded with different scan rates in the non-Faradaic region.

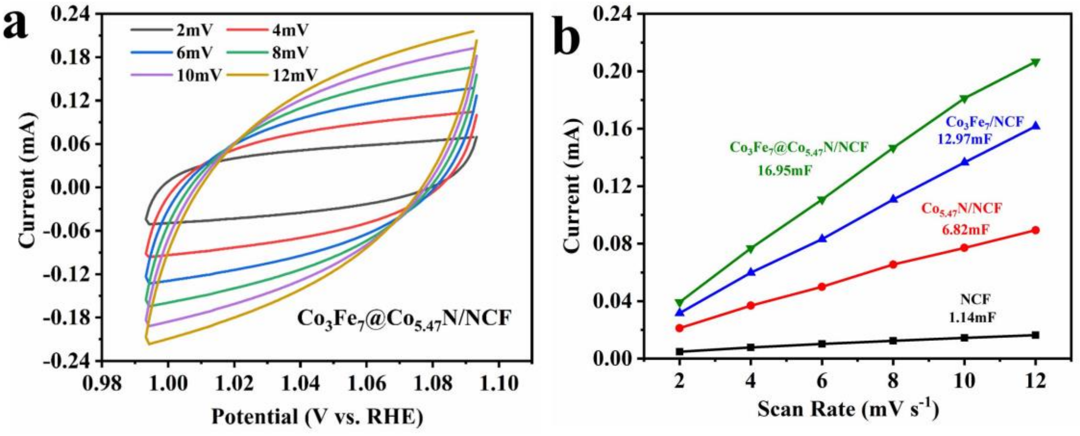

Figure S10. (a) CV curves of Co_3Fe_7@$Co_{5.47}N$/NCF at various scan rates in a 0.1 M KOH solution in the non-Faradaic region. (b) The plots of the corresponding capacitive currents as a function of scan rates ($\Delta j = (j_a - j_b)/2$) of NCF, $Co_{5.47}N$/NCF, Co_3Fe_7/NCF and Co_3Fe_7@$Co_{5.47}N$/NCF. Their C_{dll} values are given in the figure.

2. In-situ electrochemical Raman spectroscopy (EC-Raman)

In-situ electrochemical Raman spectroscopy (EC-Raman) was employed to monitor the ORR intermediates in the alkaline solution [4, 5]. The Raman spectra were recorded within the potential range from 1.11 V to 0.51 V vs. RHE with the home-made electrochemical cell (Figure S11a) using the confocal Raman spectrometer. As shown in Figure S11b, the 3D interconnected macrostructures of the $Co_3Fe_7@Co_{5.47}N/NCF$ are clearly identified and the Raman spectra were obtained from the area, which the diameter of the laser spot was 1μm.

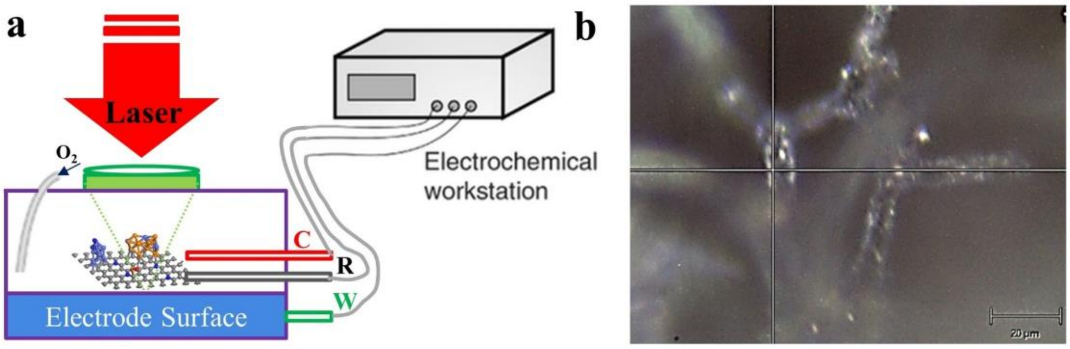

Figure S11. (a) Schematic diagram of the in situ electrochemical Raman spectroscopy experimental setup. (b) Confocal Raman images of the $Co_3Fe_7@Co_{5.47}N/NCF$.

3. Calculation Methods

DFT calculations were performed in the Vienna ab initio simulation package (VASP). A spin-polarized GGA PBE functional, all-electron plane-wave basis sets with an energy cutoff of 400 eV, and a projector augmented wave (PAW) method were adopted. Co_3Fe_7 is simulated using a surface model of p (1×1) unit cell periodicity. $Co_{5.47}N$ is simulated using a surface model of p (1×1) unit cell periodicity. A (3×3×1) Monkhorst-Pack mesh was used for the Brillouin-zone integrations to be sampled. 15 Å vacuum layer was added to avoid the interaction between adjacent layers. The conjugate gradient algorithm was used in the optimization. The convergence threshold was set $1*10^{-4}$ eV in total energy and 0.05 eV/ Å in force on each atom.

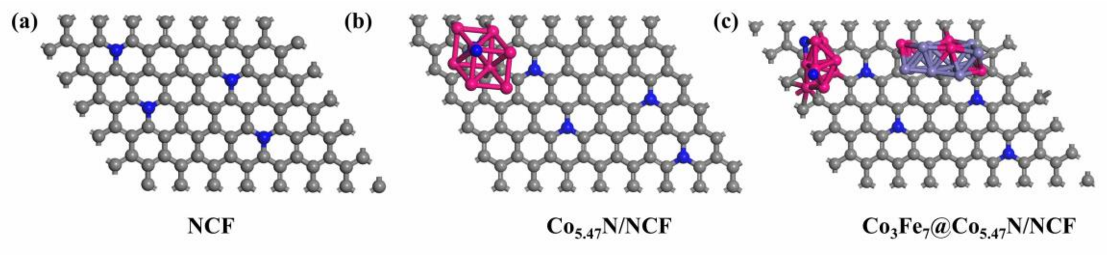

Figure S12. DFT-optimized structures of NCF, $Co_{5.47}N$/NCF and Co_3Fe_7@$Co_{5.47}N$/NCF.

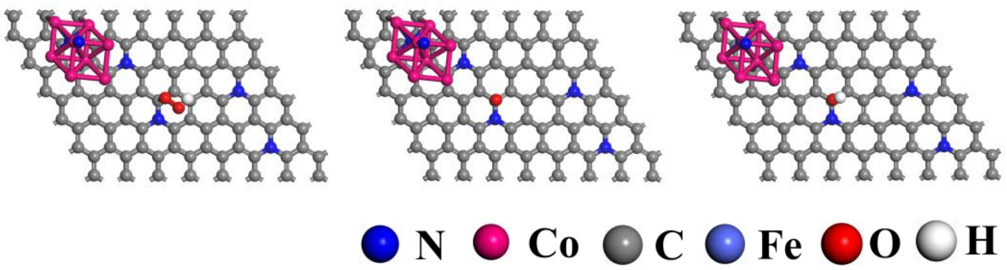

Figure S13. Optimized structures of OH*, O*, and OOH* intermediates on $Co_{5.47}N$/NCF.

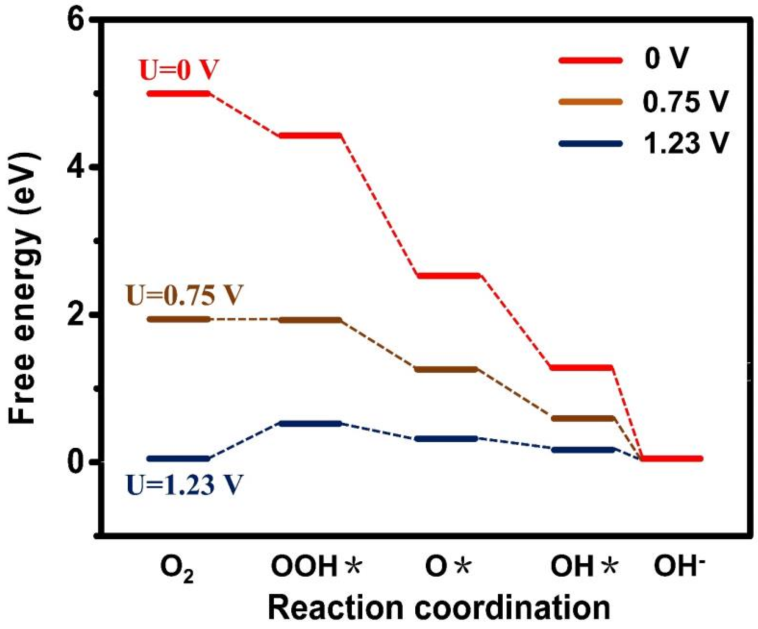

Figure S14. Free energy diagram of NCF at zero potential (U = 0), equilibrium potential (U = 1.23 V), and thermodynamic limiting potential.

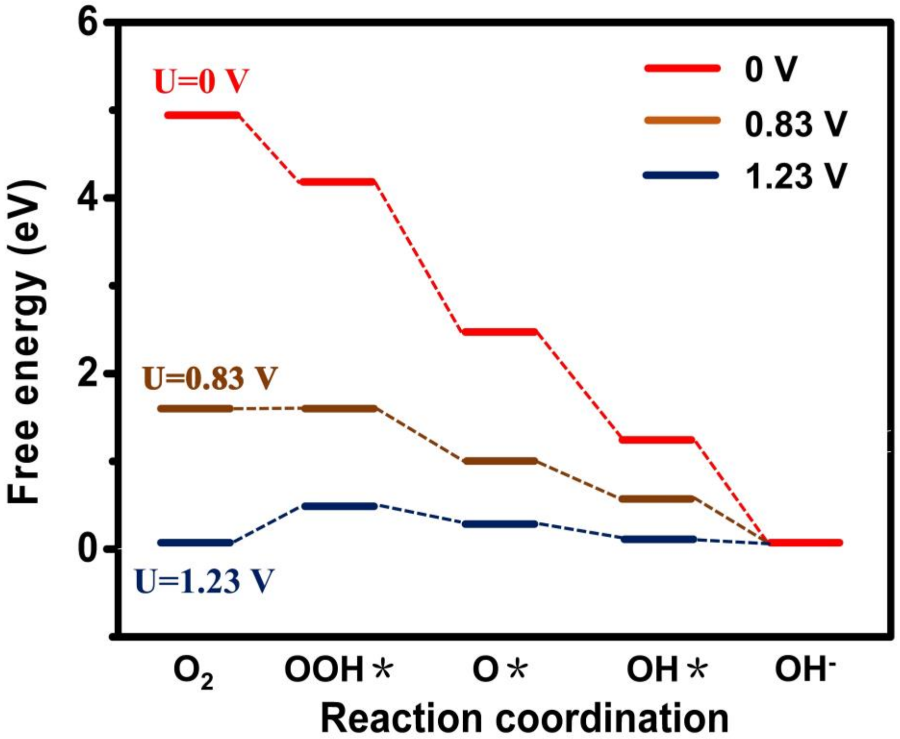

Figure S15. Free energy diagram of $Co_{5.47}N$/NCF at zero potential (U = 0), equilibrium potential (U = 1.23 V), and thermodynamic limiting potential.

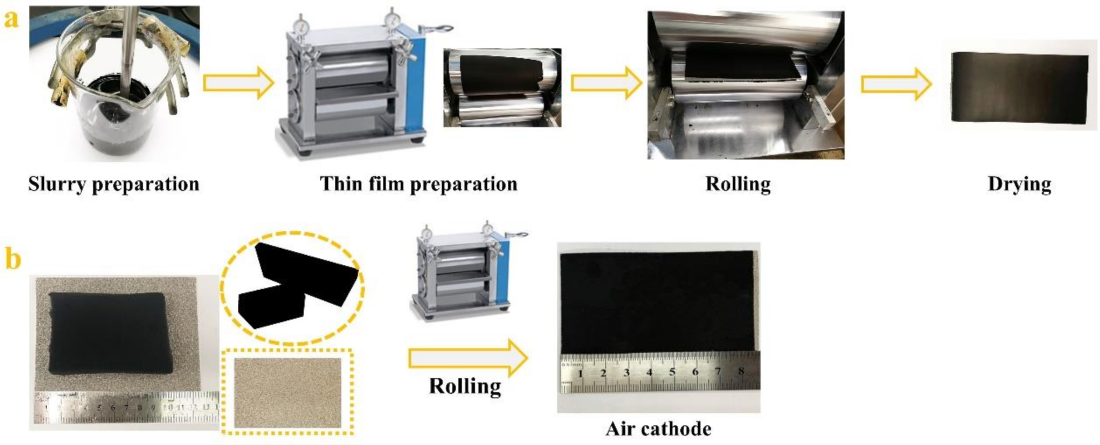

Figure S16. The methods for slurry casting cathode (a) and integrated cathode (b) of Co_3Fe_7@$Co_{5.47}N$/NCF.

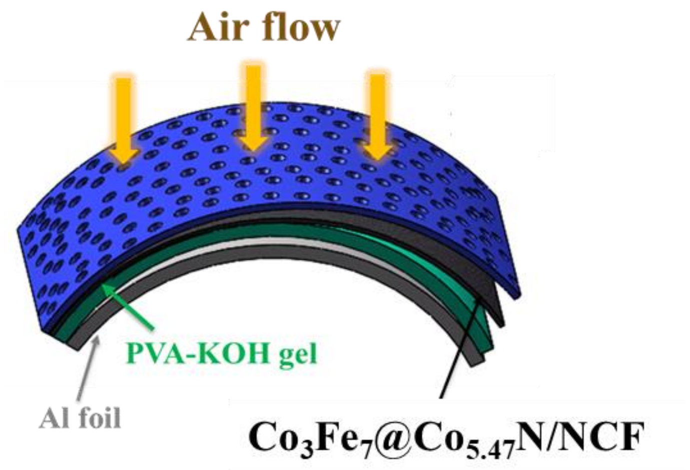

Figure S17. Illustration of the alkaline flexible Al-air battery.

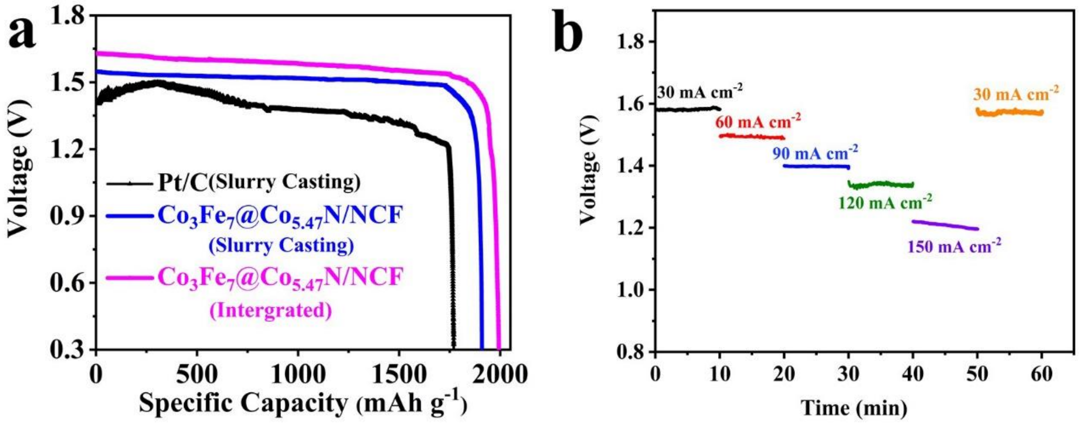

Figure S18. (a) Discharge capacities of the alkaline Al-air batteries with Co_3Fe_7@$Co_{5.47}$N/NCF integrated cathode, Co_3Fe_7@$Co_{5.47}$N/NCF slurry casting cathode and Pt/C slurry casting cathode at a current density of 20 mA cm^{-2}, (b) Rate performance of the Al-air battery with Co_3Fe_7@$Co_{5.47}$N/NCF integrated cathode.

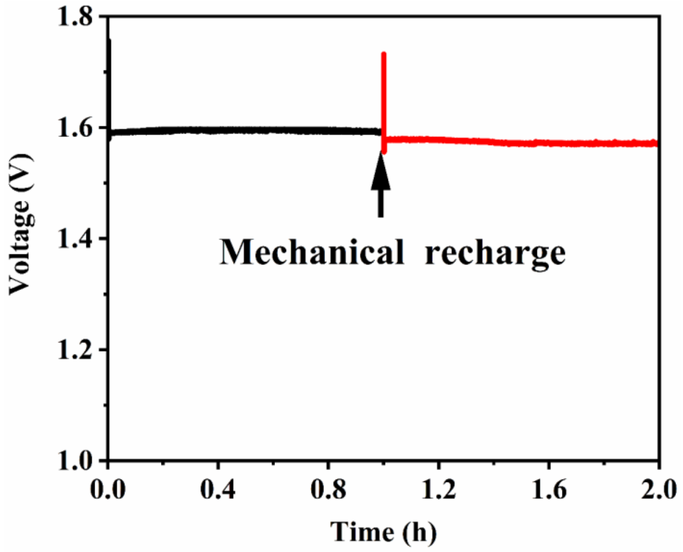

Figure S19. Discharge curves of Al-air batteries using the re-used electrolyte and a new Al anode.

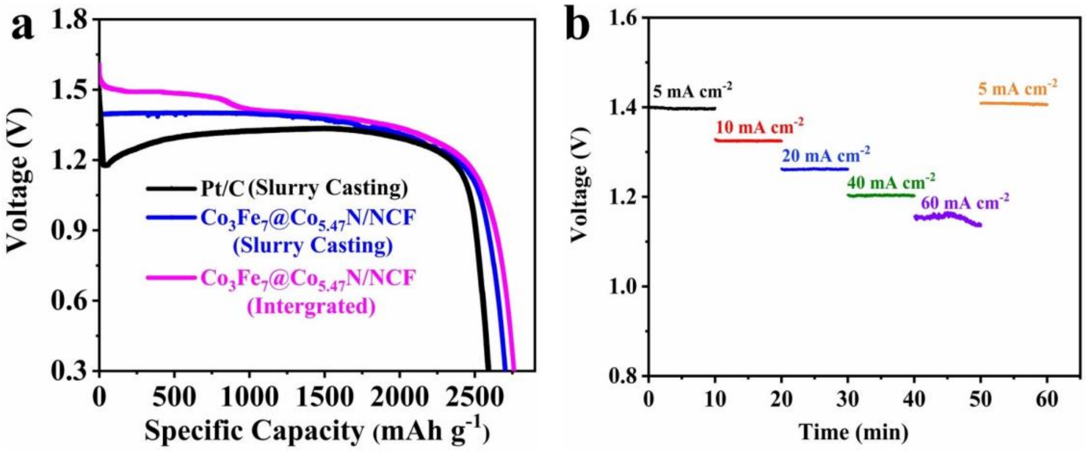

Figure S20. (a) Discharge capacities of the neutral Al-air batteries with Co_3Fe_7@$Co_{5.47}N$/NCF integrated cathode, Co_3Fe_7@$Co_{5.47}N$/NCF slurry casting cathode and Pt/C slurry casting cathode at a current density of 2 mA cm^{-2}, (b) Rate performance of the Al-air battery with Co_3Fe_7@$Co_{5.47}N$/NCF integrated cathode.

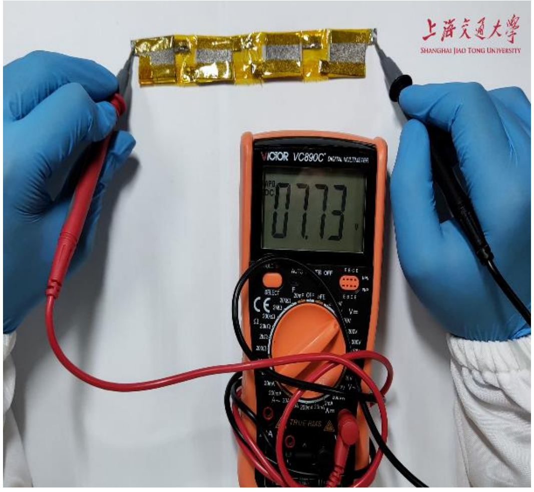

Figure S21. Open-circuit voltage of the four neutral flexible Al-air batteries in series.

Table S1 Onset and half-wave potentials of CoFe-based catalysts for ORR in 0.1 M KOH reported in recent literature

No	Catalyst Type	Onset Potential (vs. RHE)	Half-Wave Potential (vs. RHE)	Limiting Current Densities	Ref.
**1**	FeCo@MNC	0.98 V	0.86 V	5.29 mA cm^{-2}	[6]
**2**	FeCo@N-GCNT-FD	0.96 V	0.88V	6.8 mA cm^{-2}	[7]
**3**	NPSC-Co_2Fe_1	1.01 V	0.85 V	4.85 mA cm^{-2}	[8]
**4**	N-GCNT/FeCo-3	1.03 V	0.92V	4.8 mA cm^{-2}	[9]
**5**	CoC_x/FeCo@C/rGO	1.02 V	0.97 V	3.6 mA cm^{-2}	[10]
**6**	Zn_6Co_Fe ($Co_{0.9}Fe_{0.1}$)	1.03V	0.89V	5.24 mA cm^{-2}	[11]
**7**	CoNi@N-C	0.91V	0.82V	5.3 mA cm^{-2}	[12]
**8**	Co_3Fe_7/CNs-800	0.97V	0.85V	-	[13]
**9**	G-$Co_{0.6}Fe_{0.4}$	0.901V	0.80V	-	[14]
**10**	CoNi-NCF	1.02V	0.91V	5.64 mA cm^{-2}	[15]
**11**	Co_3Fe_7@$Co_{5.47}$N/NCF	1.02V	0.92V	6.3 mA cm^{-2}	This work

Table S2 Adsorption free energy of different intermediates in NCF

	O_2	OOH*	O*	OH*	OH^-
**U=0V**	4.92 eV	4.17 eV	2.62 eV	1.27 eV	0 eV
**U=0.75V**	1.92 eV	1.92 eV	1.12 eV	0.52 eV	0 eV
**U=1.23V**	0 eV	0.48 eV	0.16 eV	0.04 eV	0 eV

Table S3 Adsorption free energy of different intermediates in $Co_{5.47}N$/NCF

	**O_2**	**OOH***	**O***	**OH***	**OH^-**
**U=0V**	4.92 eV	4.09 eV	2.59 eV	1.30 eV	0 eV
**U=0.83V**	1.60 eV	1.60 eV	0.93 eV	0.47 eV	0 eV
**U=1.23V**	0 eV	0.40 eV	0.13 eV	0.07 eV	0 eV

Table S4 Adsorption free energy of different intermediates in Co_3Fe_7@$Co_{5.47}N$/NCF

	O_2	OOH*	O*	OH*	OH^-
**U=0V**	4.92 eV	3.91 eV	2.55 eV	1.25 eV	0 eV
**U=1.01V**	0.88 eV	0.88 eV	0.53 eV	0.24 eV	0 eV
**U=1.23V**	0 eV	0.22 eV	0.09 eV	0.02 eV	0 eV

Table S5. Performance of flexible Al−Air batteries reported in recent literature

S. No	Air Cathode	Electrolyte	Catalyst	Maximum Power Density	Ref.
**1**	Slurry Casting Cathode	PVA/KOH	P-CD/G	157.3 mW cm^{-2}@0.78V	[16]
**2**	Integrated Cathode	Polymer/KOH	HCA-Co-1	29.23 mW cm^{-2}@0.79V	[17]
**3**	Slurry Casting Cathode	PAM/NaCl	C-CoPAN900	3.77 mW cm^{-2}@0.77V	[18]
**4**	Slurry Casting Cathode	Filter paper/NaOH	MnO_2/CNT	43.9 mW cm^{-2}@0.70V	[19]
**5**	Slurry Casting Cathode	PAA/NaOH	MnO_2	137 mW cm^{-2}@1.20V	[20]
**6**	Slurry Casting Cathode	PAA/KOH	Fe_3C@N-CFs	49.5 mW cm^{-2}@0.61 V	[21]
**7**	Integrated Cathode	PVA/NaOH	Co_3Fe_7@$Co_{5.47}$N/NCF	199.6 mW cm^{-2}@1.00V	This work
**8**	Integrated Cathode	PVA/NaCl	Co_3Fe_7@$Co_{5.47}$N/NCF	65.0 mW cm^{-2}@0.87 V	This work

References

[1] T. Palaniselvam, V. Kashyap, S. N. Bhange, J.-B. Baek, S. Kurungot, *Adv. Funct. Mater*. **2016**, *26*, 2150.

[2] J. Guo, Y. Li, Y. Cheng, L. Dai, Z. Xiang, *ACS Nano* **2017**, *11*, 8379.

[3] C. C. L. McCrory, S. Jung, J. C. Peters, T. F. Jaramillo, *J. Am. Chem. Soc.* **2013**, *135*, 16977.

[4] J.-C. Dong, X.-G. Zhang, V. Briega-Martos, X. Jin, J. Yang, S. Chen, Z.-L. Yang, D.-Y. Wu, J. M. Feliu, C. T. Williams, Z.-Q. Tian, J.-F. Li, *Nat. Energy* **2019**, *4*, 60.

[5] M. K. Nieuwoudt, J. D. Comins, I. Cukrowski, *J. Raman Spectrosc.* **2011**, *42*, 1335.

[6] C. Li, M. Wu, R. Liu, *Appl. Catal. B-Environ.* **2019**, *244*, 150.

[7] L. An, N. Jiang, B. Li, S. Hua, Y. Fu, J. Liu, W. Hao, D. Xia, Z. Sun, *J. Mater. Chem. A* **2018**, *6*, 5962.

[8] K. He, J. Zai, X. Liu, Y. Zhu, A. Iqbal, T. Tadesse Tsega, Y. Zhang, N. Ali, X. Qian, *Appl. Catal. B-Environ.* **2020**, *265*, 118594.

[9] C.-Y. Su, H. Cheng, W. Li, Z.-Q. Liu, N. Li, Z. Hou, F.-Q. Bai, H.-X. Zhang, T.-Y. Ma, *Adv. Energy Mater.* **2017**, *7*, 1602420.

[10] H. Fang, T. Huang, Y. Sun, B. Kang, D. Liang, S. Yao, J. Yu, M. M. Dinesh, S. Wu, J. Y. Lee, S. Mao, *J Catal.* **2019**, *371*, 185.

[11] Y. Xiong, Y. Yang, F. J. DiSalvo, H. D. Abruña, *J. Am. Chem. Soc.* **2019**, *141*, 10744.

[12] H. Ning, G. Li, Y. Chen, K. Zhang, Z. Gong, R. Nie, W. Hu, Q. Xia, *ACS Appl. Mater. Inter.* **2019**, *11*, 1957.

[13] G.-L. Wen, H.-J. Niu, J.-J. Feng, X. Luo, X. Weng, A.-J. Wang, *J. Colloid Interf. Sci.* **2020**, *569*, 277.

[14] H. Khani, N. S. Grundish, D. O. Wipf, J. B. Goodenough, *Adv. Energy Mater.* **2020**, *10*, 1903215.

[15] M. Jiang, J. Yang, J. Ju, W. Zhang, L. He, J. Zhang, C. Fu, B. Sun, *Energy Storage Mater.* **2020**, *27*, 96.

[16] M. Wang, Y. Li, J. Fang, C. J. Villa, Y. Xu, S. Hao, J. Li, Y. Liu, C. Wolverton, X. Chen, V. P. Dravid, Y. Lai, *Adv. Energy Mater.* **2020**, *10*, 1902736.

[17] C. Zhu, Y. Ma, W. Zang, C. Guan, X. Liu, S. J. Pennycook, J. Wang, W. Huang, *Chem. Eng. J.* **2019**, *369*, 988.

[18] M. J. Tan, B. Li, P. Chee, X. Ge, Z. Liu, Y. Zong, X. J. Loh, *J. Power Sources* **2018**, *400*, 566.

[19] Y. Wang, H. Kwok, W. Pan, H. Zhang, D. Y. C. Leung, *J. Power Sources* **2019**, *414*, 278.

[20] Y. Sun, C. Fu, J. Ren, M. Jiang, M. Guo, R. Cheng, J. Zhang, B. Sun, *J. Electrochem. Soc.* **2020**, *167*, 080502.

[21] Y. Ma, A. Sumboja, W. Zang, S. Yin, S. Wang, S. J. Pennycook, Z. Kou, Z. Liu, X. Li, J. Wang, *ACS Appl. Mater. Inter.* **2019**, *11*, 1988.
